# Artificial Intelligence Methods in Cephalometric Image Analysis—A Systematic Narrative Review

**DOI:** 10.3390/jcm15051920

**Published:** 2026-03-03

**Authors:** Katarzyna Zaborowicz, Maciej Zaborowicz, Katarzyna Cieślińska, Barbara Biedziak

**Affiliations:** 1 Department of Orthodontics and Facial Malformations, Poznan University of Medical Sciences, Bukowska 70, 60-812 Poznań, Poland; kcieslinska@ump.edu.pl (K.C.); biedziak@ump.edu.pl (B.B.); 2 Department of Biosystems Engineering, Poznan University of Life Sciences, Wojska Polskiego 50, 60-627 Poznań, Poland

**Keywords:** artificial intelligence, cephalometric analysis, deep learning, neural networks, convolutional neural network (CNN), landmark detection, orthodontics, cervical vertebral maturation (CVM), machine learning, orthognathic surgery

## Abstract

**Background:** The dynamic development of information technologies, particularly in the fields of computer image analysis and artificial intelligence (AI) algorithms, plays an increasingly important role in orthodontic diagnostics. Cephalometric images constitute a fundamental element in orthodontic treatment planning. They contain encoded information related to the assessment of craniofacial growth and development, which is the focus of algorithms employing machine learning and process automation. **Objectives:** The aim of this paper is to present the current state of knowledge regarding the application of artificial intelligence methods in cephalometric image analysis, with particular emphasis on studies published between 2020 and 2025 in the Scopus and Web of Science databases. **Results:** Twenty key studies were included. The most commonly used models were convolutional neural networks (CNN), You Only Look Once (YOLO), Bayesian convolutional neural networks (BCNN), artificial neural networks (ANN), stacked hourglass networks, and Deep Neural Patchworks (DNP). In landmark detection tasks, the average location errors ranged from 1 to 2 mm compared to expert annotations, remaining within clinically acceptable limits. YOLO- and CNN-based systems achieved accuracy comparable to that of experienced orthodontists, while BCNN models additionally provided uncertainty estimates that improved clinical interpretability. In classification tasks, artificial neural network (ANN) models assessing cervical vertebral maturity (CVM) achieved an accuracy of up to 95%. In screening studies prior to orthognathic surgery, a multilayer perceptron combined with a regional convolutional neural network achieved 96.3% agreement with expert decisions. **Conclusions:** AI-based tools provide clinically acceptable accuracy in cephalometric analysis, with landmark detection errors typically ranging from 1 to 2 mm compared to expert assessment. These systems improve repeatability and significantly reduce analysis time, especially when used in semi-automated workflows. AI-based assessment of cervical vertebral maturity and surgical eligibility shows high agreement with expert decisions, confirming their role as reliable tools to support clinical decision-making. Nevertheless, broader validation in different patient populations is necessary before routine clinical implementation.

## 1. Introduction

Currently, there is growing interest in the application of artificial intelligence (AI) methods in various sectors of the economy and industry, but also in medicine, including the field of dentistry. AI methods are characterized by their ability to automatically learn patterns based on image data sets, which enables the analysis of complex anatomical structures without the need to manually define rules. In particular, deep learning algorithms, such as convolutional neural networks, are highly effective in detecting landmarks, achieving accuracy comparable to that of experienced clinicians. An important advantage of AI methods is the high repeatability of results and the reduction of variability resulting from subjective observer assessment, which is crucial in orthodontic diagnostics, but not only. In addition, some models, especially probabilistic and Bayesian ones, allow for the estimation of prediction uncertainty, increasing the transparency and clinical safety of decisions made. The use of AI also leads to a significant reduction in analysis time, making these methods a valuable tool to support, rather than replace, the work of a medical professional.

With the dynamic development of information technology methods and increasingly advanced image analysis tools, the importance of artificial intelligence in dental and orthodontic diagnostics is growing. Digital cephalometric images play a special role in this field, as they have been the basis for orthodontic treatment planning and the assessment of skull growth and development for many years. Widespread digitization and the wide availability of medical image databases enable the creation and refinement of machine learning models that can support clinicians in image interpretation and the automation of cephalometric measurements. For many years, cephalometric analysis has been a fundamental element of orthodontic diagnosis and treatment planning, enabling the assessment of craniofacial morphology, growth patterns, and skeletal relationships. Traditionally, it has been based on manual identification of anatomical landmarks on lateral cephalometric radiographs, which is a time-consuming process and subject to intra- and inter-observer variability. These limitations can affect the repeatability and reliability of measurements, especially in cases of complex anatomy or overlapping structures. Therefore, there is a growing need for automated and objective methods of analysis that could support clinicians by increasing the consistency of results, reducing analysis time, and limiting subjectivity in cephalometric assessment.

In recent years, prestigious and widely accessible databases such as Scopus and Web of Science have seen a dynamic increase in the number of publications on the use of artificial intelligence in cephalometric image analysis. The development of deep learning-based algorithms, including convolutional networks, has enabled the automatic identification of reference points, the performance of cephalometric measurements, and the support of malocclusion diagnosis and orthodontic treatment planning. As a result, as the authors of the cited works show, a process that was traditionally time-consuming and prone to human error has become faster, more precise, and more repeatable.

The aim of this narrative review is to present and critically analyze the most frequently cited works on the application of artificial intelligence methods in cephalometric image analysis, with particular emphasis on the detection of anatomical landmarks, assessment of cervical vertebral maturity, and prediction of orthodontic and orthognathic treatment outcomes. The analysis covered publications that could be found in the Scopus and Web of Science databases based on the keywords: “cephalometry,” “artificial intelligence,” and “reference points” [1,2,3,4,5,6,7,8,9,10,11,12,13,14,15,16,17,18,19]. The choice of these databases was methodological in nature—although some of the works are also indexed in databases dedicated to medicine, Scopus and Web of Science cover a wider spectrum of interdisciplinary journals, which potentially broadens the audience.

A proprietary methodology for selecting publications was used, based on an analysis of the most frequently cited articles published since 2020. This time frame was chosen due to a significant increase in the number of studies using deep learning techniques after 2020, which translated into dynamic development of the subject matter in question. The analysis was limited to publications in English in order to maintain a high quality of content interpretation—despite the identification of valuable works in other languages, their analysis based on translations could lead to a loss of precision in the message. The analysis was conducted until August 2025, which was related to the completion of this study in September 2025; however, it should be noted that the dynamics of citations may change. The criteria adopted are consistent with the authors’ previous review published in the Journal of Clinical Medicine and are part of a planned series of studies, which justifies their consistent unification.

## 2. Materials and Methods

This study analyzed the most frequently cited articles in the field of dentistry and artificial intelligence published in the Scopus (Table 1) and Web of Science databases between 2020 and 2025. The Scopus and Web of Science databases were selected due to their high-quality indexing. These databases were searched using the keywords: “cephalometry,” “artificial intelligence,” and “landmarks”. From each database, the 10 most frequently cited papers were selected, excluding literature reviews, short communications, and book chapters. Articles written in languages other than English were excluded from the analysis. The selected articles are open access. The analysis was conducted in August and September 2025.

Due to the narrative nature of this review, this work was not registered in the PROSPERO database. However, the literature selection process was conducted in accordance with PRISMA guidelines to ensure transparency and reproducibility. The PRISMA checklist will be included as Supplementary Material.

The analyzed data were compiled in the form of three tables. The first table (Table 2) contains information regarding the aim of the study, the number of cephalometric radiographs used, the distribution by sex, and the location where the study was conducted. The second table (Table 3) provides information on the type of neural network, test quality and error, as well as the results and conclusions drawn from the presented data. The third (Table 4) table presents the risk of bias assessment using PROBAST.

A detailed analysis and discussion of this part of the landing can be found in the Discussion Section 3.1. Discussion and Summary of Works [1,2,3,4,5,6,7,8,9,10].

The table below (Table 5) analyzes the most frequently cited articles in the field of dentistry and artificial intelligence published in the Web of Science database between 2020 and 2025.

An analysis of the presented articles was conducted, and the results are presented in the form of three tables (Table 6, Table 7 and Table 8).

A detailed analysis and discussion of this part of the landing is provided below in the Discussion Section 3.2. Discussion and Summary of Works [2,11,12,13,14,15,16,17,18,19].

## 3. Discussion

### 3.1. Discussion and Summary of Works [1,2,3,4,5,6,7,8,9,10]

When analyzing available studies on the use of artificial intelligence in orthodontics, particularly in the automatic detection of cephalometric landmarks and cervical vertebral maturity (CVM) assessment, significant differences can be observed in terms of the number of images analyzed, the accuracy of results, and consistency with expert annotations. The smallest studies were based on samples of approximately 50–100 cephalograms, e.g., in the assessment of the quality of commercial systems [6], while the largest ones included as many as 1774 images from 887 patients, including both pre- and post-treatment X-rays [8]. Several studies additionally used CBCT reconstructions (MIP), which improved the visibility of structures and the precision of landmark identification [4]. Most studies did not take gender differences into account, although analyses of skeletal growth or development emphasized the need for more representative cohorts, particularly due to developmental differences between the sexes [7].

Regarding detection precision, landmarks identified by artificial intelligence typically showed an average error of 1–2 mm compared to the expert ‘gold standard’, which is within clinically acceptable limits. Convolutional models such as YOLOv3 and multi-stage CNNs achieved an average error of approximately 1.4–1.5 mm, similar to the discrepancies observed among experts themselves [1,4]. Bayesian networks additionally provided uncertainty maps, enabling clinicians to better interpret difficult cases [2]. Particularly good results were reported for landmarks that are difficult to describe manually, where artificial intelligence sometimes outperformed human examiners [1,2]. In comparative studies, artificial intelligence and orthodontists achieved very similar results, and in semi-automatic mode (artificial intelligence + manual correction), performance was slightly higher, while significantly reducing the time needed to complete the task [9,10]. The CVM assessment performed by artificial intelligence showed high agreement with the results of experts (ICC up to 0.98) and an average annotation error of less than 0.5 mm [3].

In studies focusing on predicting treatment outcomes or planning orthognathic surgery, artificial intelligence models did not always outperform classical methods—multivariate regression or PLS methods sometimes yielded better results, while artificial intelligence performed better in soft tissue areas or anatomical areas with high variability [5,7,8]. Differences were also noted between commercial system providers—some achieved results comparable to those of experts, while others showed significant deviations, particularly in the assessment of incisor inclination [6].

### 3.2. Discussion and Summary of Works [2,11,12,13,14,15,16,17,18,19]

Analyzing available studies on the use of artificial intelligence in orthodontics—including automatic detection of cephalometric landmarks, CVM assessment, and prediction of the need for surgical treatment—significant differences can be observed in the number of images analyzed, the algorithms used, and the accuracy achieved. The sample size ranged from as few as 30–50 images in studies on new 3D methods [18,20] to over 2000 lateral cephalograms in studies on web-based solutions [13]. Growth and maturation analyses used a series of 360–419 radiographs, classified according to CVM stages [14,17]. Several studies used CBCT or CT data, which allowed for more precise localization of difficult anatomical points (e.g., gonion, foramina, and FH plane) [18,20] and even showed higher repeatability of new three-dimensional landmarks compared to conventional ones [18].

Regarding detection precision, landmarks identified by artificial intelligence in most studies showed an average error of 1–2 mm compared to expert annotations, which is within acceptable clinical standards. Convolutional models (including stacked hourglass, patch-based CNN, DNP, and BCNN) achieved errors of 1.3–1.9 mm and high SDR values (82–96% in the 2–4 mm range) [12,13,19,20], comparable to the variability between experts. Probabilistic and Bayesian models additionally enabled the estimation of uncertainty (density maps and standard ellipses) [12,19], which improved clinical interpretability, especially in cases of skeletal anomalies. Comparative studies have shown that artificial intelligence and orthodontists achieved similar results, while semi-automatic modes (artificial intelligence + expert correction) further increased accuracy and significantly reduced working time [16].

Regarding the assessment of skeletal growth and maturation (CVM), artificial neural network (ANN) models achieved better results than Bayesian classifiers, reaching an accuracy of 0.94–0.95 [14,17], while studies on sex prediction based on cervical vertebrae showed an accuracy of approximately 0.89 [14]. In the context of supporting orthognathic surgery eligibility, an MLP network integrated with the R-CNN model achieved 96.3% agreement with expert opinions [10].

The differences in results depended on the quality of data, sample size, and type of anatomical landmarks. Points on overlapping structures in the lateral projection, such as the gonion, porion, and orbitale [12,18,19], were particularly difficult to locate clearly, but artificial intelligence provided a significant improvement over conventional methods. Clinical studies have also shown variability in the accuracy of some commercial systems, especially in the assessment of incisor inclination [16]. The inclusion of 3D data and increasing the representativeness of the sample (including gender and age) have been identified as key areas for future development [18,20].

This review indicates the dynamic development of artificial intelligence applications in cephalometric analysis in recent years. Most studies confirm the clinically acceptable accuracy of automatic reference point detection, comparable to the variability observed among experienced orthodontists [1,2,4,9,13,18]. However, analysis of the studies shows that the results obtained are highly dependent on the quality of the input data, the sample size, and the type of algorithm used [4,6,13]. The scope of the research material ranged from small image collections to large, multi-center databases, which had a direct impact on the stability and generalizability of the results [6,9,13].

As is widely known, and as can be seen from the publications cited, network quality is expressed through various indicators, such as MSE, RMSE, MAE, etc. Unfortunately, to date, the system for publishing metrics for neural network models has not been unified, which significantly hinders the objective comparison and evaluation of individual models. Another issue is inferring the quality of models based on images that have been prepared by doctors with a certain degree of error (labeling errors). Even the most experienced personnel work and label images with a certain degree of accuracy. In this sense, models replicate inaccuracies in a way—this applies to networks trained using the teacher method. It can therefore be concluded that the reading of parameters contained in images depends on many aspects, including the quality and accuracy of the images, but also on the experience and accuracy of the expert.

## 4. Summary

Artificial intelligence (AI) is playing an increasingly important role in orthodontic diagnostics, particularly in the analysis of digital cephalometric images. The use of advanced algorithms enables automatic identification of reference points, assessment of cervical vertebral maturity (CVM), and prediction of orthodontic and orthognathic treatment outcomes. In recent years, numerous studies published in renowned databases such as Scopus and Web of Science have confirmed the high effectiveness of these tools.

The most commonly used artificial intelligence models include convolutional neural networks (CNN), YOLOv3, BCNN, TabNet DNN, stacked hourglass networks, Deep Neural Patchworks (DNP), and probabilistic models. In cephalogram analysis, they achieve an average detection error of 1–2 mm compared to expert annotations, which is within acceptable clinical standards. In many studies, artificial intelligence has achieved accuracy comparable to or exceeding that of orthodontists. For example, the YOLOv3 model achieved an average error of 1.46 mm [1], while Bayesian CNN (BCNN) achieved SDR rates of 82.1% (≤2 mm) and 96.0% (≤4 mm) [2], while providing uncertainty maps that increased clinical utility. DNP models enabled three-dimensional landmark detection on CT scans with errors below 2 mm, improving the repeatability and precision of orthognathic surgery planning [18].

Artificial intelligence has also been used to assess skeletal maturity and predict mandibular growth. Artificial neural networks (ANN) outperformed Bayesian classifiers, achieving an accuracy of up to 0.95 in determining CVM stages [14,17], while Lasso and Ridge methods [16] achieved the best results in predicting mandibular growth direction, with MAE errors ranging from 2.1 to 2.5 mm and ICC agreement above 0.9. In orthognathic surgery qualification, the MLP model integrated with R-CNN achieved 96.3% agreement with experts (AUC = 0.96) [10].

Research has shown that artificial intelligence systems can reduce analysis time and improve the repeatability of results. For example, automatic analysis in WebCeph™ [10] took only 10 s, and its accuracy was only slightly lower than that of the semi-automatic method, which was close to the results achieved by experts. In many cases, artificial intelligence proved to be more consistent than humans, especially for landmarks that are difficult to identify, such as the gonion or orbitals.

At the same time, researchers emphasize the need for further validation of artificial intelligence models on larger, more diverse populations, taking into account gender and age differences. The integration of 3D data (CBCT and CT) [20], the development of probabilistic models, and integration with online platforms further increase the potential of artificial intelligence in clinical practice, making it a valuable tool for orthodontists.

Despite its high effectiveness, researchers emphasize that artificial intelligence should be seen as a tool to assist specialists, not replace them. Collaboration between clinicians and algorithms allows for more accurate, faster, and more repeatable diagnostics and planning of orthodontic and surgical treatment.

This review has several limitations. Due to the narrative nature of the literature selection, there is a risk of selection bias. In our paper, we presented an overview of the most frequently cited works, noting that they were open-access works, i.e., those with potentially the widest audience reach. However, it should be noted that this may significantly affect the comprehensiveness of the review. In addition, the studies analyzed differ in terms of data set size, AI model architecture, and evaluation measures used, which limits the possibility of direct comparison of results [6,9,13,16]. The diversity of the study populations, the lack of uniform validation protocols, and different definitions of reference points further complicate the formulation of clear conclusions about the superiority of particular solutions [4,6,18]. The lack of quantitative meta-analysis and formal heterogeneity analyses was due to significant methodological and clinical heterogeneity among the included studies. The analyzed studies differed in terms of the architecture of artificial intelligence models (including proprietary neural networks), the size and structure of data sets, reference points, study populations, validation protocols, and reported outcome measures (including MRE, SDR, accuracy, and AUC). The lack of common guidelines for the design and reporting of AI-based studies makes it impossible to extract a uniform numerical effect, which is a prerequisite for conducting a reliable meta-analysis and calculating measures of statistical heterogeneity. For this reason, the synthesis of results was qualitative in nature, and the main objective of this study was to highlight the lack of standardization in terms of methodology, reporting, and the characteristics of the analyzed data sets (including the number of cases and gender distribution), which currently limits the possibility of directly comparing results between studies.

No formal sensitivity analyses were performed either, as the lack of uniform, comparable effect indicators made it impossible to assess the stability of the aggregated results in quantitative terms. An additional limitation was the inconsistency in the reporting of confidence intervals and precision measures in the analyzed publications, which limits the possibility of direct comparison. These limitations reflect the broader problem of the lack of uniform methodological and reporting standards in studies using artificial intelligence, which poses a significant challenge for future review and meta-analytic work.

After analyzing the work, it should be noted that the quality of the model depends heavily on the data set (images) used in the modeling process. The quality of models depends on the algorithms themselves, their complexity, and the learning method (supervised learning with a teacher or unsupervised learning without a teacher). Currently, artificial intelligence methods used in scientific research are widely tested. The number of studies and the use of large training sets in the future will allow us to determine the appropriate methods for specific problems. Neural modeling is used where it is difficult to apply classical methods, i.e., traditional computer algorithms. The use of these methods seems to be good for solving nonlinear problems. Due to the above-mentioned characteristics, AI methods are suitable for this type of problem and can handle it at the level of expert knowledge or better.

## 5. Conclusions

Artificial intelligence is becoming an increasingly important element of modern orthodontic diagnostics, especially in the field of cephalometric analysis of digital images. A review of studies published between 2020 and 2025 indicates that machine learning and deep learning algorithms enable automatic localization of reference points with clinically acceptable accuracy, comparable to the variability between experienced orthodontists. AI systems demonstrate high repeatability and significantly reduce analysis time, and their application also includes the assessment of cervical vertebral maturity and the prediction of growth and orthodontic and orthognathic treatment outcomes.

Despite promising results, the further development and implementation of artificial intelligence into everyday clinical practice requires the standardization of research methods, the validation of models on larger and more diverse populations, and the wider integration of three-dimensional data. Artificial intelligence should be seen as a tool to assist the clinician, not replace them, enabling increased efficiency, repeatability, and quality of orthodontic diagnostics.

## Figures and Tables

**Table 1:** The selected articles from the Scopus database [1,2,3,4,5,6,7,8,9,10].

Number	Title	First Author	Journal	Year of Publication	Number of Citations
1	Automated identification of cephalometric landmarks: Part 2—Might it be better than human? [1]	Hye-Won Hwang	Angle Orthodontist	2020	183
2	Automated cephalometric landmark detection with confidence regions using Bayesian convolutional neural networks [2]	Jeong-Hoon Lee	BMC Oral Health	2020	112
3	Development of an Artificial Intelligence System for the Automatic Evaluation of Cervical Vertebral Maturation Status [3]	Jing Zhou	Diagnostics	2021	41
4	Automatic Cephalometric Landmark Identification System Based on the Multi-Stage Convolutional Neural Networks with CBCT Combination Images [4]	Min-Jung Kim	Sensors	2021	27
5	Does artificial intelligence predict orthognathic surgical outcomes better than conventional linear regression methods? [5]	Ji-Ae Park	Angle Orthodontist	2024	11
6	Assessment of the quality of different commercial providers using artificial intelligence for automated cephalometric analysis compared to human orthodontic experts [6]	Felix Kunz	Journal of Orofacial Orthopedics	2025	10
7	Prediction of Pubertal Mandibular Growth in Males with Class II Malocclusion by Utilizing Machine Learning [7]	Grant Zakhar	Diagnostics	2023	9
8	Orthodontic treatment outcome predictive performance differences between artificial intelligence and conventional methods [8]	Sung Joo Cho	Angle Orthodontist	2024	7
9	Human examination and artificial intelligence in cephalometric landmark detection—is AI ready to take over? [9]	Suvarna Indermun	Dentomaxillofacial Radiology	2023	7
10	Reliability and accuracy of artificial intelligence-based software for cephalometric diagnosis: A diagnostic study [10]	Francesca Assi	Angle Orthodontist	2022	6

**Table 2:** The Scopus database: the aim of the study, the number of cephalometric radiographs, the gender distribution, and the location [1,2,3,4,5,6,7,8,9,10].

Number	Research Objective	Total Images	Gender Distribution	Study Location
1	Comparison of the detection of 80 cephalometric landmarks by an automatic identification system based on the deep learning method (YOLOv3) with the detection of these landmarks by human experts, in terms of both accuracy and repeatability	1311: 1028 training + 283 testing.	Training: 507 F (49.3%), 521 M (50.7%). Testing: 146 F (51.6%), 137 M (48.4%).	Seoul National University Dental Hospital and Seoul National University School of Dentistry and Dental Research Institute, Seoul, Korea
2	Development of a framework for an automatic cephalometric landmark detection system with 95% confidence regions using Bayesian Convolutional Neural Networks (BCNN) to improve accuracy, reliability, and clinical utility	400	Not specified	Ewha Womans University Medical Center, Seoul, and Yonsei University, Seoul, Korea
3	Development of an artificial intelligence system (CNN) for automatic evaluation of cervical vertebral maturation (CVM) and comparison of its performance with human assessment in terms of accuracy and repeatability	1080: 980 training, 100 testing.	Training: 432 M, 548 F. Testing: 43 M, 57 F.	West China Hospital of Stomatology, Sichuan University, Chengdu, China
4	Development and validation of a fully automated cephalometric landmark identification system based on multi-stage Convolutional Neural Networks (multi-stage CNNs), utilizing combined conventional lateral cephalograms synthesized from CBCT and Maximum Intensity Projection (MIP) images	430 CBCT scans → 860 images (430 CBCT-LC + 430 MIP-LC)	Not specified	Kyung Hee University Dental Hospital, Seoul, Korea
5	Evaluation of the effectiveness of an artificial intelligence model (TabNet deep neural network) in predicting orthognathic surgical outcomes compared to traditional linear regression methods (MLR and PLS)	1410	392 F (55.6%), 313 M (44.4%)	Seoul National University Dental Hospital and Seoul National University School of Dentistry, Korea
6	Assessment of the quality and accuracy of cephalometric analyses generated by four commercial AI providers (DentaliQ.ortho, WebCeph, AudaxCeph, CephX) compared to the “gold standard” established by orthodontic experts	50	Not specified	Department of Orthodontics, University Hospital of Würzburg and University Hospital of Regensburg, Germany
7	Development and evaluation of machine learning models for predicting the direction and magnitude of mandibular growth during puberty in boys with Class II malocclusion	369	Males only	Indiana University School of Dentistry, Indianapolis, USA
8	Evaluation of the effectiveness of an artificial intelligence model (TabNet DNN) in predicting soft tissue and alveolar bone changes after orthodontic treatment, compared to conventional prediction methods (MMLR and PLSR)	1774	604 F (68.1%), 283 M (31.9%)	Department of Orthodontics, Seoul National University Dental Hospital and Seoul National University School of Dentistry, Korea
9	Comparison of the precision of two cephalometric landmark identification methods: (1) computer-assisted human assessment (Dolphin Imaging^®^), (2) AI-based program (BoneFinder^®^), on data from the South African population	409	237 F (57.94%), 172 M (42.05%)	Department of Craniofacial Biology, Pathology and Radiology, and Department of Orthodontics, University of the Western Cape, Cape Town; data from Tygerberg Oral Health Center, South Africa
10	Evaluation of the reliability, accuracy, and processing time of AI software for cephalometric analysis (automatic and semi-automatic modes in WebCeph™) compared to conventional digital tracing (NemoCeph™) on 2D lateral cephalograms	408	241 F (59.1%), 167 M (40.9%)	Universidad Alfonso X el Sabio, Madrid, Spain

**Table 3:** The Scopus database: the type of neural network, test quality and error and conclusions [1,2,3,4,5,6,7,8,9,10].

Number	Type of Neural Network	Test Quality and Error	Research Findings
1	Deep Neural Network—YOLOv3 (You-Only-Look-Once, v3)	Mean detection error: AI vs. human: 1.46 ± 2.97 mm Human vs. human: 1.50 ± 1.48 mm Intra-examiner repeatability: 0.97 ± 1.03 mm	AI achieved accuracy comparable to orthodontists, with most differences <0.9 mm (clinically insignificant). AI showed perfect repeatability and was not significantly affected by image quality or metal artifacts.
2	Bayesian Convolutional Neural Network (BCNN)	Mean detection error: Landmark error (LE): 1.53 ± 1.74 mm SDR: 82.1% (≤2 mm), 92.3% (≤3 mm), 96.0% (≤4 mm)	BCNN achieved higher precision than previous methods, especially for Gonion (nearly halved the error). The use of 95% confidence regions improved clinical interpretability and educational utility.
3	Convolutional Neural Network (CNN—DetNet based on ResNet50)	Mean detection error: AI vs. human: 0.36 ± 0.09 mm Human vs. human: 0.48 ± 0.12 mm Classification accuracy for CVM stages: 71% (best for CS6: F1 = 85%, CS1 = 77%; weakest for CS3: F1 = 31%)	AI–expert agreement was very high (ICC = 0.98); AI error was lower than inter-examiner error.
4	Multi-Stage Convolutional Neural Network (CNN)	Mean radial error (MRE): 1.03 ± 1.29 mm SDR: 87.1% (≤2 mm), 91.2% (≤2.5 mm), 93.5% (≤3 mm), 96.6% (≤4 mm)	Highest accuracy for Nasion (0.56 mm) and Pogonion (0.58 mm); lowest for Gonion (2.04 mm). Eight of fifteen landmarks had <1 mm error. Comparable performance on CBCT-LC and MIP-LC images.
5	TabNet Deep Neural Network (DNN)—LOOCV validation	Compared to MLR (lowest), PLS, and AI (TabNet). Prediction error: mean Euclidean distance between predicted vs. actual soft-tissue landmark positions. PLS better for 16 points, AI better for 6 points, no significant difference for 10 points.	AI better captured postoperative variability in lower jaw & neck; PLS was better for stable facial areas. Combining AI and PLS was recommended for optimal surgical outcome prediction.
6	Commercial AI systems: DentaliQ.ortho, WebCeph, AudaxCeph, CephX	DentaliQ.ortho—no significant differences in 9/9 parameters vs. experts. WebCeph—no significant differences but lower precision. AudaxCeph—significant deviations in 7/9 parameters. CephX—significant deviations in 5/9 parameters (up to ~6°). All systems less precise for incisor inclination.	DentaliQ.ortho showed best agreement with experts; CephX performed worst. All systems were time-efficient but should be used under clinical supervision.
7	Machine Learning models—XGBoost, Random Forest, Lasso, Ridge, Linear Regression, SVR, MLP	2-year prediction: MAE mandibular length: 2.11–6.07 mm (best: Lasso; worst: Linear Regression) MAE *Y*-axis: 0.85–2.74° ICC: 0.90–0.95; Accuracy: 97–99% 4-year prediction: MAE mandibular length: 2.32–5.28 mm MAE *Y*-axis: 1.25–1.72° ICC: 0.76–0.87; Accuracy: 96–98%	ML predicted mandibular length within ~2.5 mm and growth direction within ~1°. Lasso and Ridge were best; Linear Regression was worst. Key predictors: age, facial height, incisor position & inclination, angles SN-MP, SN-Pog, SNB, SNA.
8	TabNet Deep Neural Network (DNN)	Pooled average prediction error: MMLR: 1.69 mm PLSR: 1.74 mm AI (TabNet): 2.12 mm AI superior in only 5/44 landmarks (11.4%), all in soft tissue below menton–neck.	MMLR achieved highest accuracy overall, especially for bony & alveolar landmarks. AI was better only for soft-tissue neck points.
9	Fully Automatic Landmark Annotation (FALA)—BoneFinder^®^	Mean Euclidean distance vs. Dolphin Imaging^®^ within 2–4 mm. High intra-/inter-examiner repeatability (ICC > 0.9). AI repeatability perfect (r = 1.0) in internal tests.	BoneFinder^®^ accuracy was comparable to Dolphin Imaging^®^, with most errors within 2–4 mm. Perfect repeatability outperformed human examiners. Largest discrepancies for Gonion and Orbitale.
10	Deep Neural Network—WebCeph™ (AI based on deep learning)	Mean deviations vs. conventional method: Automatic AI: 0.06–1.17°/mm (clinically significant in SNA, Wits, U1-NA°, L1-NB°, IMPA) Semi-automatic AI: 0.06–1.17°/mm (clinically significant only in L1-NB°)	Automatic AI was fastest (~10 s); semi-automatic AI (~2.5 min) was most accurate and closest to expert results; conventional method >9 min.

**Table 4:** The Scopus database: the risk of bias [1,2,3,4,5,6,7,8,9,10].

Study	Participants	Predictors	Outcome	Analysis	Overall Risk of Bias
Hwang et al. (2020) [1]	Low risk	Low risk	Unclear	High risk	High
Lee et al. (2020) [2]	Unclear	Low risk	Unclear	High risk	High
Zhou et al. (2021) [3]	Some concern	Low risk	Some concern	High risk	High
Kim et al. (2021) [4]	Unclear	Low risk	Unclear	High risk	High
Park et al. (2024) [5]	Some concern	Low risk	Low risk	High risk	High
Kunz et al. (2023/25) [6]	High risk	Low risk	Some concern	High risk	High
Zakhar et al. (2023) [7]	High risk	Low risk	Low risk	High risk	High
Cho et al. (2024) [8]	Some concern	Low risk	Low risk	High risk	High
Indermun et al. (2023) [9]	Some concern	Low risk	High risk	Some concern	High
Mercier et al. (2024) [10]	Unclear	Low risk	Unclear	High risk	High

**Table 5:** The selected articles from the Web of Science database [2,11,12,13,14,15,16,17,18,19].

Number	Title	First Author	Journal	Year of Publication	Number of Citations
1	Automated cephalometric landmark detection with confidence regions using Bayesian convolutional neural networks [2]	Jeong-Hoon Lee	BMC Oral Health	2020	103
2	Web-based fully automated cephalometric analysis by deep learning [11]	Hannah Kim	Computer Methods and Programs in Biomedicine	2020	91
3	Determination of growth and development periods in orthodontics with artificial neural network [12]	Hatice Kök	Orthodontics & Craniofacial Research	2021	35
4	Artificial intelligence-based cephalometric landmark annotation and measurements according to Arnett’s analysis [13]	Thaísa P. Silva	Dentomaxillofacial Radiology	2022	20
5	Preciseness of artificial intelligence for lateral cephalometric measurements [14]	Mostafa El-Dawlatly	Journal of Orofacial Orthopedics	2024	15
6	Evaluation of the Artificial Neural Network and Naive Bayes Models Trained with Vertebra Ratios for Growth and Development Determination [15]	Hatice Kök	Turkish Journal of Orthodontics	2021	14
7	Three-Dimensional Cephalometric Landmarking and Frankfort Horizontal Plane Construction: Reproducibility of Conventional and Novel Landmarks [16]	Gauthier Dot	Journal of Clinical Medicine	2021	13
8	Multistage Probabilistic Approach for the Localization of Cephalometric Landmarks [17]	Hyuk Jin Kwon	IEEE Access	2021	11
9	Automated detection of cephalometric landmarks using deep neural patchworks [18]	Julia Vera Weingart	Dentomaxillofacial Radiology	2023	9
10	Application of a Multi-Layer Perceptron in Preoperative Screening for Orthognathic Surgery [19]	Natkritta Chaiprasittikul	Healthcare Informatics Research	2023	8

**Table 6:** The Web of Science database: the aim of the study, the number of cephalometric radiographs, the gender distribution, and the location [2,11,12,13,14,15,16,17,18,19].

Number	Research Objective	Total Images	Gender Distribution	Study Location
1	Development of a framework for an automatic cephalometric landmark detection system with 95% confidence regions using Bayesian Convolutional Neural Networks (BCNN) to improve accuracy and reliability	ISBI 2015 dataset (exact number not reported)	Not specified	Ewha Womans University Medical Center and Yonsei University, Seoul, Korea
2	Development of a fully web-based automated cephalometric analysis system using a stacked-hourglass model for detecting 23 landmarks	2075 lateral cephalograms from 2 institutions	Not specified	Korea Institute of Science and Technology; Korea University of Science & Technology; Yonsei University.; Korea University. Anam Hospital; Chung-Ang University Hospital; Seoul National University (SNU), Korea
3	Determination of growth and development stages based on cervical vertebrae using an Artificial Neural Network (ANN)	419 cephalograms + wrist radiographs	6 age groups; each with 35 girls and 35 boys (total: 210 F, 209 M)	Selçuk University, Konya, Turkey
4	Evaluation of the reliability of CEFBOT AI software for automatic landmark identification and Arnett analysis measurements	30 lateral cephalograms	Not specified	Federal University of Sergipe and Federal University of Bahia, Brazil
5	Evaluation of the accuracy and efficiency of AI in cephalometric measurements (comparison of WebCeph AI, modified AI, and manual analysis in OnyxCeph)	200 lateral cephalograms	Not specified	Faculty of Dentistry, Cairo University, Egypt
6	Assessment of the performance of ANN and Naive Bayes models in predicting growth and development stages based on cervical vertebrae ratios	360 cephalograms divided into 6 groups (60 subjects each)	Each group: 30 girls and 30 boys	Selçuk University, Konya, Turkey
7	Assessment of the repeatability and reproducibility of 3D landmark identification and Frankfurt plane construction using both conventional and novel (foraminal, dental) landmarks	20 CT scans of orthognathic surgery patients	Not specified	Arts et Métiers ParisTech; Université de Paris; AP-HP Pitié-Salpêtrière Hospital, France
8	Development of a multi-stage probabilistic CNN-based method for cephalometric landmark localization, incorporating both global and local features	ISBI 2015 dataset (exact number not reported)	Not specified	Seoul National University and Ajou University, Korea
9	Evaluation of the accuracy of Deep Neural Patchworks (DNP) for automatic identification of 60 cephalometric landmarks on CT scans for orthodontic and orthognathic treatment planning	30 full CT scans (15 training, 15 testing)	18 F (60%), 12 M (40%)	University Medical Center Freiburg, Germany
10	Development of a system to assist in screening for orthognathic surgery eligibility using a Multilayer Perceptron (MLP) and R-CNN	538 digital lateral cephalograms	Not specified	Mahidol University and Srinakharinwirot University, Thailand

**Table 7:** The Web of Science database: the type of neural network, test quality and error, and conclusions [2,11,12,13,14,15,16,17,18,19].

Number	Type of Neural Network	Test Quality and Error	Research Findings
1	Bayesian Convolutional Neural Network (BCNN)—cephalometric landmark detection with confidence regions	Mean landmark error (LE): 1.53 ± 1.74 mm SDR: 82.1% (≤2 mm), 92.3% (≤3 mm), 96.0% (≤4 mm)	Compared to previous methods, the error was especially reduced for difficult landmarks (e.g., Gonion—almost halved). The model provided 95% confidence regions, improving clinical and educational utility.
2	Stacked Hourglass CNN—Web-based fully automated cephalometric analysis	Mean point-to-point error: 1.37 ± 1.79 mm (23 landmarks) Automatic anatomical type classification accuracy: 88.4%	The model generalized well across devices and institutions, enabling fully web-based automated cephalometric analysis and reducing diagnostic time.
3	Artificial Neural Network (ANN)—growth stage and sex assessment based on cervical vertebrae	ANN-7 (32 measures + age): accuracy = 0.9427 Best simplified model (13 measures): accuracy = 0.8687 Sex identification from vertebrae: accuracy = 0.8950	ANN accurately classified CVM stages and sex based on cephalometric images, showing potential as a clinical decision-support system.
4	AI-based CEFBOT—automatic annotation and Arnett analysis measurements	ICC > 0.94 for 8/9 measurements Lowest repeatability for FH-THL angle: ICC = 0.768 No significant differences between AI and expert for other measurements	CEFBOT demonstrated high reliability and reproducibility (except for one angle) and may improve efficiency in clinical radiological practice.
5	WebCeph—AI vs. AI with manual correction vs. OnyxCeph (manual reference standard)	Significant differences between three methods Closest results: AI + manual correction vs. OnyxCeph AI alone fastest but less accurate	Combining AI with manual correction yielded the highest accuracy, whereas AI alone was fastest but less reliable for clinical purposes.
6	Artificial Neural Network (ANN) vs. Naive Bayes (NB)—growth stage prediction from cervical vertebrae ratios	Best: ANN-3 = 0.95; weakest: NB-4 = 0.50 Slightly lower accuracy for stage 5 (CVM): 0.83	ANN models outperformed NB. Key predictors were ratios of upper and anterior borders and cervical vertebral depth.
7	Machine Learning models: XGBoost, Random Forest, Lasso, Ridge, Linear Regression, SVR, MLP	2-year prediction: MAE mandibular length: 2.11–6.07 mm (best Lasso, worst Linear Regression) MAE *Y*-axis: 0.85–2.74° ICC: 0.90–0.95; Accuracy: 97–99% 4-year prediction: MAE mandibular length: 2.32–5.28 mm MAE *Y*-axis: 1.25–1.72° ICC: 0.76–0.87; Accuracy: 96–98%	ML predicted mandibular length within ~2.5 mm and growth direction within ~1°. Lasso and Ridge performed best; Linear Regression was worst. Key predictors: age, facial height (upper & lower), incisor position & inclination, angles SN-MP, SN-Pog, SNB, SNA.
8	Multistage Probabilistic CNN—cephalometric landmark localization	Trained on ISBI 2015 dataset Combined global features with local image patches Additional correction for mandibular landmarks	Multistage approach improved accuracy, particularly for bilateral mandibular landmarks (e.g., Gonion), and allowed for prediction confidence regions.
9	Deep Neural Patchworks (DNP)—automatic 3D landmark detection on CT	Mean AI error: 1.94 mm (SD 1.45) vs. manual: 1.32 mm (SD 1.08) Lowest errors for ANS (1.11 mm), SN (1.20 mm), CP_R (1.25 mm)	DNP enabled automatic detection of 60 3D cephalometric landmarks on CT with <2 mm error, enhancing reproducibility and planning in orthodontics and orthognathic surgery.
10	Multi-Layer Perceptron (MLP) + Keypoint R-CNN—pre-screening for orthognathic surgery	Dataset: 538 digital cephalograms 7 input variables AI–expert decision agreement: 96.3% AUC = 0.96, sensitivity = 1, precision = 0.93	The AI tool effectively supports decision-making for surgical eligibility, potentially facilitating referrals and shortening qualification time.

**Table 8:** The Web of Science database: the risk of bias [2,11,12,13,14,15,16,17,18,19].

Study	Participants	Predictors	Outcome	Analysis	Overall Risk of Bias
Lee et al. (2020) [2]	Unclear	Low risk	Unclear	High risk	High
Kim et al. (2020) [11]	Some concern	Low risk	Unclear	High risk	High
Kök et al. (2021) [12]	Some concern	Low risk	Some concern	High risk	High
Silva et al. (2022) [13]	Unclear	Low risk	Some concern	Some concern	High
El-Dawlatly et al. (2023) [14]	Some concern	Low risk	Some concern	High risk	High
Kök et al. (2021) [15]	Some concern	Low risk	Some concern	High risk	High
Dot et al. (2021) [16]	Some concern	Low risk	Some concern	High risk	High
Kwon et al. (2021) [17]	Unclear	Low risk	Unclear	High risk	High
Weingart et al. (2023) [18]	Some concern	Low risk	Some concern	High risk	High
Chaiprasittikul et al. (2023) [19]	Some concern	Low risk	Low risk	High risk	High

## Data Availability

No new data were created or analyzed in this study.

## Supplementary Materials

The following supporting information can be downloaded at: https://www.mdpi.com/article/10.3390/jcm15051920/s1, Supplementary Material S1: PRISMA 2020 Checklist [21].

## Abbreviations

The following abbreviations are used in this manuscript:

AI	Artificial Intelligence
ANS	Anterior Nasal Spine
AUC	Area Under the Curve
CNN	Convolutional Neural Network
CBCT	Cone Beam Computed Tomography
CP_R	Condylion Posterior – Right
BCNN	Bayesian Convolutional Neural Network
ANN	Artificial Neural Network
DNP	Digital Nerve Processing
CVM	Computer Vision Model
DNN	Deep Neural Network
FH–THL	FH – Frankfort Horizontal plane – THL – True Horizontal Line
SN	Sella–Nasion line
SN–MP	Sella–Nasion to Mandibular Plane angle
SN–Pog	Sella–Nasion to Pogonion angle
SNB	Sella–Nasion–B point angle
SNA	Sella–Nasion–A point angle
SVR	Support Vector Regression
SDR	Successful Detection Rate
MLP	Multilayer Perceptron
MAE	Mean Absolute Error
ML	Machine Learning
MLR	Multiple Linear Regression
MMLR	Multivariate Multiple Linear Regression
MSE	Mean Squared Error
MIP	Maximum Intensity Projection
PLS	Partial Least Squares Regression
PLSR	Partial Least Squares Regression
R-CNN	Region-based Convolutional Neural Network
RMSE	Root Mean Squared Error
U1–NA°	Upper incisor to NA angle
L1–NB°	Lower incisor to NB angle
IMPA	Incisor Mandibular Plane Angle
ISBI	International Symposium on Biomedical Imaging
YOLO	You Only Look Once
ICC	Intraclass Correlation Coefficient
