# Supplementary material for: Artificial Intelligence Methods in Cephalometric Image Analysis—A Systematic Narrative Review
Source: J Clin Med. 2026 Mar 3;15(5):1920. doi: 10.3390/jcm15051920 (PMC12986449; doi:10.3390/jcm15051920)
Supplement: Supplementary file 1 [file jcm-15-01920-s001.zip › jcm-4062818-supplementary.pdf]

PRISMA 2020 Checklist

Section and Topic	Item #	Checklist item	Location where item is reported
TITLE			
Title	1	Identify the report as a systematic review.<br>In a review examining the use of artificial intelligence methods in the analysis of cephalometric images, the authors identify the report as a systematic review: “Artificial intelligence in cephalometric image analysis – a systematic review.”	Section: Title Page number: 1 Line numbers: 1–3
ABSTRACT			
Abstract	2	See the PRISMA 2020 for Abstracts checklist.<br>In a review examining the use of artificial intelligence methods in the analysis of cephalometric images, the authors summarise the objectives, eligibility criteria, databases consulted, methods for data collection, evaluation of diagnostic model performance, and synthesis of results, presenting key outcomes and highlighting the limitations of available evidence. They also specify that the review was not funded and has no registered protocol.<br>Abstract: Artificial intelligence (AI) plays an increasingly important role in orthodontic diagnostics, particularly in the analysis of digital cephalometric images. The aim of this review was to present the current state of knowledge on the use of AI methods in cephalometric image analysis, focusing on studies indexed in the Scopus and Web of Science databases from 2020 to 2025. The databases were searched using the terms “cephalometry,” “artificial intelligence,” and “landmarks,” and the ten most frequently cited original research papers from each database were included. Literature reviews, short communications, and non-English articles were excluded. The review presents data on the number of cephalometric images analysed, neural network types, accuracy metrics, and bias assessment using PROBAST. The results indicate that convolutional neural networks (CNNs), YOLOv3, TabNet, and Bayesian CNNs achieved mean detection errors of 1–2 mm, comparable to human experts. AI systems demonstrated high repeatability and significantly reduced analysis time. Some studies also explored AI for growth assessment and orthognathic surgery prediction, achieving over 90% accuracy. Despite its high effectiveness, the authors emphasise that AI should assist clinicians rather than replace them, and further validation on diverse populations is necessary. The review received no external funding and was not registered in a review protocol.	Section: Abstract Page number: 1 Line numbers: 11–39
INTRODUCTION			
Rationale	3	Describe the rationale for the review in the context of existing knowledge.<br>In a review examining the application of artificial intelligence methods in cephalometric image analysis, the authors recognise the rapid advancement of information technology and image processing tools in dentistry and orthodontics, as well as the increasing importance of AI in diagnostic procedures. They note that, although numerous studies have been published on the use of AI for detecting cephalometric landmarks and assessing skeletal development, no systematic synthesis of these findings has been conducted in recent years.	Section: Introduction<br>Page number: 2<br>Line numbers: 44–65
Objectives	4	Provide an explicit statement of the objective(s) or question(s) the review addresses.<br>In a review examining the use of artificial intelligence methods in cephalometric image analysis, the authors clearly define the main objective of their work:<br>Objective: To present the current state of knowledge on the use of artificial intelligence methods in cephalometric image analysis, with particular emphasis on studies available in the Scopus and Web of Science databases from 2020 to 2025. The review aimed to evaluate (1) the accuracy and repeatability of AI-based landmark detection, (2) the effectiveness of AI models in assessing cervical vertebral maturation (CVM) and predicting orthodontic or orthognathic treatment outcomes, and (3) the comparability of AI system performance with expert clinicians. Additionally, the review sought to identify current limitations and directions for further development of AI applications in orthodontic diagnostics.	Section: Introduction<br>Page number: 2<br>Line numbers: 66–70
METHODS			

PRISMA 2020 Checklist

Section and Topic	Item #	Checklist item	Location where item is reported
Eligibility criteria	5	Specify the inclusion and exclusion criteria for the review and how studies were grouped for the syntheses.<br>In a review examining the use of artificial intelligence methods in cephalometric image analysis, the authors specify detailed inclusion and exclusion criteria concerning study type, diagnostic scope, methodology, and publication characteristics. The criteria are clearly outlined in the Materials and Methods section and supported by tabulated data.<br>Inclusion criteria: (1) studies focusing on the use of artificial intelligence for cephalometric landmark detection, cervical vertebral maturation (CVM) assessment, or prediction of orthodontic and orthognathic treatment outcomes; (2) original research articles indexed in the Scopus and Web of Science databases; (3) studies using AI-based approaches such as convolutional neural networks (CNN), YOLOv3, Bayesian CNN, TabNet, or other machine learning algorithms; (4) studies providing quantitative performance metrics (e.g., detection error, ICC, SDR, MAE, or accuracy); (5) publications from 2020–2025; and (6) articles written in English and available in open access.<br>Exclusion criteria: literature reviews, short communications, book chapters, and studies not employing artificial intelligence or not using cephalometric images.<br>The selected studies were grouped for synthesis according to their primary objectives: (1) automatic landmark detection, (2) skeletal maturation and growth prediction, (3) orthognathic treatment outcome prediction, and (4) evaluation of commercial AI systems. The results were summarised in tables presenting study aims, datasets, neural network types, test quality and error metrics, findings, and bias assessments (Tables 1–8).	Section: Materials and Methods Page number: 2 Line numbers: 72–84
Information sources	6	Specify all databases, registers, websites, organisations, reference lists and other sources searched or consulted to identify studies. Specify the date when each source was last searched or consulted.<br>In a review examining the application of artificial intelligence methods in cephalometric image analysis, the authors clearly identify the electronic databases searched, the search period, and the keywords used to locate eligible studies, as well as the exclusion of certain publication types:<br>We conducted an electronic search for eligible studies in the Scopus and Web of Science databases, covering the period from 2020 to 2025. The searches were performed in August and September 2025. The databases were chosen due to their high indexing quality and broad coverage of international scientific publications in dentistry and medical imaging. Studies were identified using the following keywords: ‘cephalometry’, ‘artificial intelligence’, and ‘landmarks’.<br>From each database, the ten most frequently cited original research articles were selected. Literature reviews, short communications, and book chapters were excluded, as were articles written in languages other than English. Only open-access studies meeting the inclusion criteria were analysed. The review did not include searches of reference lists, registers, or additional databases beyond Scopus and Web of Science.	Section: Materials and Methods Page number: 2 Line numbers: 72–76
Search strategy	7	Present the full search strategies for all databases, registers and websites, including any filters and limits used.<br>In a review examining the application of artificial intelligence methods in cephalometric image analysis, the authors provide a detailed description of the search strategy applied to both databases used. The following excerpt summarises the full search strategy for Scopus and Web of Science:<br>We used the advanced search interfaces of the Scopus and Web of Science databases and conducted systematic searches in August and September 2025, covering publications from 2020 to 2025.<br>The search was carried out using predefined keyword combinations relevant to the field of orthodontic diagnostics and artificial intelligence:<br>• ‘cephalometry’ AND ‘artificial intelligence’ AND ‘landmarks’<br>No additional Boolean operators, truncations, or filters were applied beyond the date range (2020–2025) and language restriction (English).<br>To ensure high-quality and representative results, only original research articles were included. Literature reviews, short communications, and book chapters were excluded. Articles not written in English or without open-access availability were also omitted.<br>Search results were organised separately for each database. From both Scopus and Web of Science, the ten most frequently cited original studies were selected for inclusion. The final set of analysed papers and their characteristics are presented in Tables 1–8.	Section: Materials and Methods Page number: 2 Line numbers: 77–80
Selection process	8	Specify the methods used to decide whether a study met the inclusion criteria of the review, including how many reviewers screened each record and each report retrieved, whether they worked independently, and if applicable, details of automation tools used in the process.<br>In a review examining the application of artificial intelligence methods in cephalometric image analysis, the authors describe a structured and independent screening process to ensure the accuracy and reliability of study selection:<br>Initially, the authors conducted a pilot review of a subset of articles retrieved from the Scopus and Web of Science databases to verify the clarity	Section: Materials and Methods Page number: 2

Section and Topic	Item #	Checklist item	Location where item is reported
		and consistency of the inclusion and exclusion criteria. Then the titles and abstracts were screened of all records identified in the search to determine eligibility according to the predefined criteria.<br>Any disagreements regarding study eligibility were resolved through discussion and consensus. In cases where consensus could not be achieved, a third reviewer was consulted to make the final decision. No automation tools or machine-assisted screening systems were used in the selection process. The final set of included studies comprised the ten most frequently cited original articles from each database that met all inclusion criteria.	Line numbers: 72–84
Data collection process	9	Specify the methods used to collect data from reports, including how many reviewers collected data from each report, whether they worked independently, any processes for obtaining or confirming data from study investigators, and if applicable, details of automation tools used in the process.<br>In a review examining the application of artificial intelligence methods in cephalometric image analysis, the authors describe a systematic data extraction process performed independently by two reviewers, using structured tables to ensure completeness and consistency:<br>A standardized data extraction form was developed to collect relevant information from each included study. Extracted data included: study objective, number of cephalometric images analysed, gender distribution, study location, type of neural network or AI model used, evaluation metrics (e.g., detection error, SDR, ICC, MAE), and main research findings.<br>The collected information was summarised and presented in tabular form (Tables 1–8), categorised according to the type of analysis (e.g., landmark detection, growth assessment, treatment prediction, and system comparison). The authors did not contact original study investigators for clarification or additional data, and no automation tools were used in the data extraction process.	Section: Materials and Methods Page number: 2 Line numbers: 72-84
Data items	10a	List and define all outcomes for which data were sought. Specify whether all results that were compatible with each outcome domain in each study were sought (e.g. for all measures, time points, analyses), and if not, the methods used to decide which results to collect.<br>In a review examining the application of artificial intelligence methods in cephalometric image analysis, the authors clearly define the outcome domains of interest, the corresponding quantitative measures, and the approach used to select results when multiple outcomes were reported:<br>Eligible outcomes were grouped into three primary analytical domains related to cephalometric diagnostics:<br>• Automatic landmark detection – defined as the ability of AI models to automatically identify cephalometric reference points on 2D or 3D images. Reported outcomes included mean detection error (mm), success detection rate (SDR) within defined error ranges (≤2 mm, ≤3 mm, ≤4 mm), and intra-/inter-examiner agreement (ICC).<br>• Skeletal maturation and growth prediction (CVM analysis) – defined as the use of AI models (e.g., CNN, ANN, Bayesian networks) to classify cervical vertebral maturation stages or to predict mandibular growth. Reported outcomes included classification accuracy, mean absolute error (MAE), and correlation coefficients (ICC).<br>• Treatment outcome prediction and system comparison – defined as AI-assisted prediction of orthodontic or orthognathic treatment outcomes or performance comparison among commercial AI software systems. Reported metrics included prediction error (mm or degrees), accuracy, sensitivity, and agreement with human experts.<br>All studies were required to report at least one quantitative measure of model performance (e.g., detection error, SDR, ICC, accuracy, or MAE). When multiple performance indicators were available, the most clinically relevant and comprehensive results were extracted — prioritising overall accuracy and SDR within ≤2 mm or ≤3 mm error thresholds.<br>If a study compared several AI models or neural network architectures (e.g., YOLOv3, BCNN, TabNet, DNP), each model's performance was recorded separately. When multiple datasets or experimental configurations were presented, results from the best-performing or most clinically applicable setup were selected for synthesis. Subgroup data (e.g., by gender or age) were included only when providing distinct insights into model performance.	Section: Materials and Methods; Tables 1–8 Page numbers: 2–12
	10b	List and define all other variables for which data were sought (e.g. participant and intervention characteristics, funding sources). Describe any assumptions made about any missing or unclear information.<br>Participant characteristics:<br>Gender distribution – recorded for each study when reported (e.g., number or percentage of male and female participants). However, several	Section: Materials and Methods; Tables 1–8

Section and Topic	Item #	Checklist item	Location where item is reported
		studies did not specify gender data, particularly in those using anonymised or secondary image databases.<br>Patient age – inconsistently reported; when unavailable, participant age was inferred from study context (e.g., pediatric, adolescent, or adult populations) or based on the stated objective, such as growth prediction studies involving pubertal males.<br>Study location – extracted from each publication and reported as the country or specific institution (e.g., Seoul National University, West China Hospital of Stomatology, Indiana University).<br>Intervention characteristics (AI models):<br>Type of neural network or machine learning algorithm – recorded for each study (e.g., YOLOv3, CNN, Bayesian CNN, TabNet DNN, Multi-Stage CNN, Deep Neural Patchworks, or MLP).<br>Performance metrics – included quantitative parameters describing model accuracy and test quality, such as mean detection error (mm), success detection rate (SDR), intra-class correlation coefficient (ICC), mean absolute error (MAE), or classification accuracy (%).<br>Number of cephalometric radiographs or scans – recorded as total images analysed, including training and testing subsets (ranging from fewer than 50 to over 1,700 images).<br>Scope of analysis – categorised according to diagnostic purpose: (1) automatic cephalometric landmark detection, (2) assessment of cervical vertebral maturation (CVM) or skeletal growth, (3) prediction of orthodontic or orthognathic treatment outcomes, and (4) comparison of commercial AI systems.<br>Funding sources:<br>The authors explicitly stated that “this study did not receive external funding.”<br>Assumptions for missing or unclear data:<br>When specific participant details (e.g., gender, age) or technical parameters (e.g., training/testing split, neural network depth) were not reported, the information was marked as “not specified” in the synthesis tables. If methodological context allowed reasonable inference—such as recognising a dataset as public (e.g., ISBI 2015) or an institution-specific database—the likely characteristics were noted. No imputation methods or automation tools were used to address missing data.	Page numbers: 2–12
Study risk of bias assessment	11	Specify the methods used to assess risk of bias in the included studies, including details of the tool(s) used, how many reviewers assessed each study and whether they worked independently, and if applicable, details of automation tools used in the process.<br>In the review examining the application of artificial intelligence methods in cephalometric image analysis, the authors report that the risk of bias assessment was conducted using the PROBAST (Prediction model Risk Of Bias ASsessment Tool). The evaluation included four domains: participants, predictors, outcomes, and analysis.<br>Each included study was assessed and classified as having low risk, some concern, or high risk in each domain. The overall risk of bias was determined based on these ratings, and the results were summarised in tabular form (Tables 4 and 8).<br>Two reviewers independently evaluated each study according to the PROBAST criteria. Discrepancies in assessments were resolved through discussion and consensus; no automation tools were used in the process.<br>The results of the bias assessment indicated that the majority of studies were rated as having a high overall risk of bias, primarily due to methodological heterogeneity, limited dataset sizes, and unclear reporting of predictor and outcome selection.	Section: Materials and Methods; Tables 4 and 8<br>Page numbers: 7 and 12
Effect measures	12	Specify for each outcome the effect measure(s) (e.g. risk ratio, mean difference) used in the synthesis or presentation of results.<br>In the review examining the application of artificial intelligence methods in cephalometric image analysis, the authors describe how they handled outcome data across studies employing diverse AI models, datasets, and evaluation criteria:<br>Because the included studies reported a wide range of quantitative performance indicators, we used standardised effect measures to enable comparison across different analytical domains.	Section: Materials and Methods; Tables 3, 7<br>Page

Section and Topic	Item #	Checklist item	Location where item is reported
		For automatic landmark detection, outcomes were expressed using:<br>Mean detection error (mm) – representing the average Euclidean distance between AI-predicted and expert-annotated landmarks.<br>Success detection rate (SDR) – reported as the percentage of landmarks detected within thresholds of ≤2 mm, ≤3 mm, or ≤4 mm.<br>Intraclass correlation coefficient (ICC) – indicating agreement between AI and human examiners.<br>For skeletal growth and cervical vertebral maturation (CVM) assessment, the main effect measures included:<br>Classification accuracy (%), mean absolute error (MAE), and ICC between AI predictions and human ratings.<br>For treatment outcome prediction and comparative evaluation of commercial systems, results were presented as:<br>Prediction error (mm or degrees), accuracy (%), and correlation with clinical measurements.<br>Given the heterogeneity of models, imaging modalities, and evaluation frameworks, no single summary statistic (e.g., pooled mean difference or risk ratio) was calculated. Instead, results were synthesised descriptively, and effect sizes were presented in tabular form for each study domain (Tables 3 and 7). When multiple measures were reported, the most clinically relevant and comprehensive indicators (e.g., SDR and ICC) were prioritised for inclusion.	numbers: 5 and 10-11
Synthesis methods	13a	Describe the processes used to decide which studies were eligible for each synthesis (e.g. tabulating the study intervention characteristics and comparing against the planned groups for each synthesis (item #5)).<br>In the review examining the application of artificial intelligence methods in cephalometric image analysis, the authors explain that the included studies were organised into predefined analytical categories based on their primary research objectives and methodological characteristics:<br>Given the diversity of AI applications and methodological approaches across the included studies, we categorised them into four main analytical domains corresponding to the objectives outlined in the review:<br>Automatic cephalometric landmark detection – studies evaluating AI models for detecting and localising cephalometric reference points.<br>Skeletal maturation and growth prediction (CVM analysis) – studies assessing AI systems for cervical vertebral maturation staging or mandibular growth prediction.<br>Orthodontic and orthognathic treatment outcome prediction – studies comparing AI-based predictive models with conventional statistical approaches.<br>Evaluation of commercial AI systems for cephalometric analysis – studies benchmarking proprietary AI tools (e.g., WebCeph™, DentaliQ.ortho, CephX) against expert tracings.<br>To determine eligibility for each synthesis, the authors tabulated the key characteristics of all included studies, including:<br>AI model type (e.g., CNN, YOLOv3, BCNN, TabNet, MLP, DNP);<br>Dataset attributes (number of cephalograms or CBCT images, training/testing split, image dimensionality);<br>Performance metrics (e.g., mean detection error, SDR, ICC, MAE, accuracy); and<br>Study setting (institution, country, or clinical context).<br>Each study was then allocated to the synthesis group that best reflected its main analytical aim. If a study addressed multiple objectives (e.g., both landmark detection and growth prediction), it was assigned to the category that represented its primary focus as indicated by the study authors or its title.<br>The grouped results were then summarised in dedicated tables (Tables 1–8), each corresponding to a synthesis category, to ensure clarity, comparability, and methodological consistency across analyses.	Section: Materials and Methods Page number: 2-12 Line numbers: 72-174
	13b	Describe any methods required to prepare the data for presentation or synthesis, such as handling of missing summary statistics, or data	Section:

Section and Topic	Item #	Checklist item	Location where item is reported
		conversions.<br>In the review examining the application of artificial intelligence methods in cephalometric image analysis, the authors describe how extracted data were standardised and prepared for synthesis across studies that used diverse AI models, datasets, and reporting formats:<br>Due to variations in reporting styles and performance metrics among the included studies, we performed several data preparation and standardisation steps prior to synthesis and tabulation.<br>When studies reported detection accuracy or error in different units (e.g., pixels, millimetres, or degrees), all values were converted into millimetres (mm) or degrees to allow direct comparison of performance outcomes across models. For studies using success detection rates (SDR) within multiple thresholds (≤2 mm, ≤3 mm, ≤4 mm), we consistently extracted the SDR ≤2 mm value as the primary measure of model precision.<br>If studies provided multiple AI configurations or model versions (e.g., YOLOv3 vs. Multi-Stage CNN or different CNN architectures), only the best-performing version—defined by the lowest mean detection error or highest SDR/ICC—was included in the synthesis tables.<br>When key statistics such as standard deviation (SD) or ICC confidence intervals were missing, these were not estimated but marked as "not reported." No imputation or extrapolation procedures were applied to reconstruct missing values.<br>All extracted data were organised into structured summary tables (Tables 1–8), grouped by analysis type (e.g., landmark detection, CVM assessment, growth prediction, system comparison). No automated tools were used in data cleaning or transformation; all conversions and selections were performed manually by two reviewers to ensure consistency and accuracy of presentation.	Materials and Methods Page number: 2-12 Line numbers: 72-174
	13c	Describe any methods used to tabulate or visually display results of individual studies and syntheses.<br>In the review examining the application of artificial intelligence methods in cephalometric image analysis, the authors report using detailed, standardised tables to present the characteristics, methodologies, and findings of individual studies, organised according to the analytical focus of each synthesis:<br>We created structured summary tables to systematically and visually present the key characteristics and outcomes of each included study. Each table contains the following data elements: study objective, total number of cephalometric images (including training and testing subsets), gender distribution (if reported), research location or institution, type of neural network used, evaluation metrics (e.g., mean detection error, SDR, ICC, MAE, accuracy), and the main conclusions drawn by the study authors.<br>To enhance clarity and comparability, separate tables were prepared for each synthesis category:<br>Tables 1–3: studies from the Scopus database (landmark detection, model type, results and error analysis).<br>Tables 5–7: studies from the Web of Science database (growth assessment, treatment prediction, and 3D analyses).<br>Each set of tables corresponds to a specific analytical domain—automatic landmark detection, skeletal maturation and growth prediction, treatment outcome prediction, or evaluation of commercial AI systems.<br>Visual presentation was standardised to allow readers to easily compare AI model performance across studies and identify trends in accuracy, repeatability, and clinical applicability. The use of multi-column tables allowed the alignment of performance metrics and error measures for direct comparison between different algorithms (e.g., YOLOv3 vs. BCNN vs. TabNet).<br>All data were presented in tabular rather than graphical form, as this format best captured detailed numerical metrics and methodological differences between studies. No automated or graphical visualisation tools were employed; all tables were manually compiled to ensure consistency and completeness.	Section: Materials and Methods Page number: 2-12 Line numbers: 72-174
	13d	Describe any methods used to synthesize results and provide a rationale for the choice(s). If meta-analysis was performed, describe the model(s), method(s) to identify the presence and extent of statistical heterogeneity, and software package(s) used.<br>In the review examining the application of artificial intelligence methods in cephalometric image analysis, the authors explain that, due to methodological and technical heterogeneity among the included studies, a narrative (qualitative) synthesis was conducted instead of a meta-analysis:	Section: Materials and Methods Page number: 2-12

Section and Topic	Item #	Checklist item	Location where item is reported
		Given the considerable heterogeneity across studies — including variations in AI architectures (e.g., YOLOv3, BCNN, TabNet DNN, CNN, Multi-Stage CNN, MLP), dataset size and quality, imaging modalities (2D cephalograms, CBCT, CT-derived reconstructions), and reporting metrics (e.g., mean detection error, SDR, ICC, MAE, accuracy) — quantitative pooling of results was not feasible. Therefore, a narrative synthesis of findings was performed.<br>The synthesis was organised by analytical domain, corresponding to the main objectives of the review:<br>Automatic cephalometric landmark detection.<br>Skeletal maturation and growth prediction (CVM analysis).<br>Orthodontic and orthognathic treatment outcome prediction.<br>Evaluation of commercial AI systems.<br>For each synthesis category, results were summarised and compared descriptively using structured tables that presented the study objectives, sample sizes, model types, performance metrics, and main findings. When studies reported results for multiple AI models or configurations, only the best-performing version (with the lowest detection error or highest SDR/accuracy) was included in the synthesis to ensure interpretive consistency.<br>No meta-analysis was performed, and consequently, no statistical models or heterogeneity measures (e.g., I^2, Q-test) were applied. Likewise, no statistical software (e.g., RevMan, STATA, or R) was used for data aggregation.<br>The rationale for adopting a narrative synthesis was to maintain methodological transparency and accurately reflect the substantial variability in study design, data quality, and AI model implementation, which precluded meaningful quantitative comparison. The synthesis therefore relied on qualitative assessment supported by tabular summaries (Tables 1–8) to identify overarching trends and performance patterns across studies.	Line numbers: 72-174
	13e	Describe any methods used to explore possible causes of heterogeneity among study results (e.g. subgroup analysis, meta-regression).<br>In the review examining the application of artificial intelligence methods in cephalometric image analysis, the authors report that—owing to methodological diversity among studies—they explored possible sources of heterogeneity through structured qualitative comparisons rather than formal statistical analyses:<br>Because of substantial heterogeneity in AI architectures, dataset composition, imaging modalities, and reported outcome metrics, we investigated potential sources of variation in study results through predefined subgroup comparisons.<br>Studies were grouped and compared according to:<br>Type of AI model used (e.g., YOLOv3, CNN, BCNN, TabNet, Multi-Stage CNN, DNP, or MLP).<br>Size and nature of the dataset (e.g., studies using fewer than 500 cephalograms vs. large datasets exceeding 1,000 images; 2D cephalograms vs. 3D CBCT or CT-based datasets).<br>Analytical objective (automatic landmark detection, skeletal maturation and growth prediction, treatment outcome prediction, or evaluation of commercial AI systems).<br>Performance evaluation metrics (e.g., mean detection error, SDR, ICC, MAE, classification accuracy).<br>These subgroup comparisons were established a priori as likely contributors to differences in reported accuracy and repeatability among studies, based on the variability identified during the selection and data extraction phases.<br>Reported performance metrics were then qualitatively compared within and across subgroups to identify consistent patterns — for example, the observation that convolutional models such as YOLOv3 and multi-stage CNNs generally achieved higher accuracy (mean error ~1.4–1.5 mm) than traditional single-layer networks, or that larger datasets were associated with improved model generalisability.<br>Because of the heterogeneity in study design, performance measures, and reporting standards, formal meta-regression or statistical heterogeneity analyses (e.g., I^2) were not performed. Instead, this structured comparative approach allowed for descriptive interpretation of	Section: Materials and Methods Page number: 2-12 Line numbers: 72-174

Section and Topic	Item #	Checklist item	Location where item is reported
		methodological factors contributing to variability in AI performance across the included studies.	
	13f	Describe any sensitivity analyses conducted to assess robustness of the synthesized results.<br>In the review examining the application of artificial intelligence methods in cephalometric image analysis, the authors report performing qualitative sensitivity analyses to verify the robustness and consistency of the synthesized findings across varying study designs and data characteristics:<br>No formal statistical sensitivity analysis or re-estimation of pooled effect sizes was conducted, as the review employed a narrative synthesis. However, these structured comparisons confirmed that the general trends in diagnostic accuracy and repeatability—particularly for convolutional neural networks and large datasets—remained consistent across all analytical conditions.	Section: Materials and Methods Page number: 2-12 Line numbers: 72-174
Reporting bias assessment	14	Describe any methods used to assess risk of bias due to missing results in a synthesis (arising from reporting biases).<br>In the review examining the application of artificial intelligence methods in cephalometric image analysis, the authors describe the qualitative procedures used to identify potential reporting or publication bias across the included studies:<br>Because the number of comparable studies within each analytical category (e.g., landmark detection, growth prediction, treatment outcome prediction) was limited, no statistical methods such as funnel plots or Egger's tests were applied to assess publication bias. The heterogeneity of performance metrics and the diversity of AI model architectures made such quantitative bias assessment impractical.<br>To evaluate the risk of selective outcome reporting, we compared the study objectives and analytical scope described in the introduction and methods sections of each article with the performance metrics actually reported in the results. If key indicators—such as mean detection error, SDR, or ICC—were mentioned as evaluation measures but not included in the results, the study was classified as having a *potential risk of reporting bias.*<br>Additionally, the abstracts and full texts of the included papers were cross-checked to ensure that performance outcomes were consistently presented across reporting levels. Inconsistencies, such as overstated performance claims in abstracts or missing data in tables, were noted qualitatively.<br>No formal statistical or automated bias detection tools were used. Instead, this manual comparative approach provided a qualitative assessment of potential reporting bias within the synthesis. The limited number of eligible studies and the lack of registered protocols for most primary research further contributed to an overall *moderate risk of reporting bias* across the analysed evidence.	Section: Materials and Methods; Tables 4 and 8 Page numbers: 7 and 12
Certainty assessment	15	Describe any methods used to assess certainty (or confidence) in the body of evidence for an outcome.<br>In the review examining the application of artificial intelligence methods in cephalometric image analysis, the authors outline a qualitative approach to assessing the certainty and reliability of the body of evidence for each analytical category:<br>We expressed the level of confidence in the synthesized findings using consistent and transparent language, reflecting how much trust can be placed in the results for each main outcome domain — automatic landmark detection, skeletal maturation and growth prediction, treatment outcome prediction, and evaluation of commercial AI systems.<br>When assessing certainty in the evidence, the following factors were considered:<br>Quality and completeness of reported performance data – including whether key quantitative metrics such as mean detection error, SDR, ICC, MAE, or accuracy were provided in full.<br>Dataset size and representativeness – higher confidence was assigned to studies using large, diverse datasets (typically >1,000 images) or validated open-access databases; lower confidence was assigned to small-scale or single-centre studies.<br>Transparency of AI methodology – confidence increased when the type and configuration of the neural network, training/testing split, and validation procedures were clearly described; it decreased when models (especially commercial systems) lacked methodological detail.<br>Reproducibility and comparability – studies reporting cross-validation results or benchmarking against expert tracings were considered more	Section: Materials and Methods Page number: 2-12 Line numbers: 72-174

Section and Topic	Item #	Checklist item	Location where item is reported
		reliable than those without such comparisons.<br>Overall, greater confidence was placed in studies that used large, well-documented datasets, clearly described their AI architectures and validation methods, and presented comprehensive quantitative results. Lower confidence was assigned to studies with incomplete reporting, non-transparent algorithms, or limited sample diversity.<br>No formal grading framework (such as GRADE) was applied; instead, this structured qualitative approach allowed for a transparent and reasoned judgment of the evidence strength in each synthesis domain.	
RESULTS			
Study selection	16a	Describe the results of the search and selection process, from the number of records identified in the search to the number of studies included in the review, ideally using a flow diagram.<br>In the review examining the application of artificial intelligence methods in cephalometric image analysis, the authors describe a systematic and transparent process of study identification and selection based on predefined eligibility criteria:<br>The literature search was conducted in the Scopus and Web of Science databases between August and September 2025, covering the publication period from 2020 to 2025. The search strategy used the keywords *'cephalometry,' 'artificial intelligence,'* and *'landmarks'.*<br>The initial search yielded numerous records in both databases. After removing duplicates and screening titles and abstracts, studies were evaluated according to the inclusion and exclusion criteria — only original research papers written in English, using artificial intelligence methods for cephalometric image analysis, were retained. Literature reviews, short communications, book chapters, and non-English publications were excluded.<br>From each database, the ten most frequently cited eligible studies were selected, resulting in a total of twenty original research articles included in the final synthesis (10 from Scopus and 10 from Web of Science).	Section: Materials and Methods Page number: 2-12 Line numbers: 72-174
	16b	Cite studies that might appear to meet the inclusion criteria, but which were excluded, and explain why they were excluded.<br>In the review examining the application of artificial intelligence methods in cephalometric image analysis, the authors indicate that several studies initially appeared to meet the inclusion criteria but were ultimately excluded after full-text assessment. The exclusions were based on predefined methodological and linguistic criteria:<br>A total of 20 original studies were included in the final synthesis (10 from Scopus and 10 from Web of Science). During the selection process, a number of records were excluded for the following reasons:<br>Study type: review articles, short communications, book chapters, and conference abstracts were excluded because they did not provide original research data or performance metrics of AI models.<br>Imaging modality: some potentially relevant studies were excluded because they used non-cephalometric imaging techniques (e.g., panoramic or CBCT-only datasets) rather than lateral cephalograms.<br>Methodological limitations: studies that did not apply artificial intelligence or machine learning algorithms to cephalometric analysis, or that lacked quantitative performance indicators (e.g., mean detection error, SDR, ICC), were excluded.<br>Language of publication: articles not written in English were omitted, even when their abstracts suggested relevance to the review topic.<br>Accessibility: studies not available in open access were excluded to maintain transparency and replicability of the review process.<br>Although the authors clearly state these exclusion criteria, no formal table of excluded studies or specific citations was provided in the review. However, they note that several publications identified during the search phase were removed for the above reasons before compiling the final dataset.<br>Consequently, while certain works might have initially appeared to meet the inclusion criteria based on title or abstract, they were excluded primarily because of differences in imaging modality, lack of quantitative data, or non-English publication language.	Section: Materials and Methods<br>- Page: 2<br>- Text lines: 72-80
Study	17	Cite each included study and present its characteristics.	Section:

Section and Topic	Item #	Checklist item	Location where item is reported
characteristics		In the review examining the application of artificial intelligence methods in cephalometric image analysis, the authors present detailed characteristics of all included studies in structured tabular format. These tables (Tables 1–8) summarise the most frequently cited and methodologically significant works identified from the Scopus and Web of Science databases, covering the years 2020–2025.<br>A comprehensive summary of the main characteristics of the included studies is provided in Tables 1–8, corresponding to the two primary databases (Scopus and Web of Science) and four analytical domains: automatic landmark detection, skeletal maturation and growth prediction, treatment outcome prediction, and evaluation of commercial AI systems. Each table includes the following elements:<br>Author(s) and year of publication;<br>Study title and journal;<br>Research location and institution;<br>Sample size (number of cephalometric images, training/testing distribution, and gender breakdown, if reported);<br>Type of AI model or neural network architecture used (e.g., YOLOv3, Bayesian CNN, TabNet DNN, Multi-Stage CNN, Deep Neural Patchworks, MLP);<br>Performance metrics (e.g., mean detection error, SDR, ICC, MAE, accuracy); and<br>Main findings and clinical conclusions drawn by the study authors.<br>The following 20 original studies were included in the review:<br>From Scopus (2020–2025):<br>Hye-Won Hwang et al. (2020) – *Automated identification of cephalometric landmarks: Part 2 – Might it be better than human? Angle Orthodontist.*<br>Jeong-Hoon Lee et al. (2020) – *Automated cephalometric landmark detection with confidence regions using Bayesian convolutional neural networks. BMC Oral Health.*<br>Jing Zhou et al. (2021) – *Development of an Artificial Intelligence System for the Automatic Evaluation of Cervical Vertebral Maturation Status. Diagnostics.*<br>Min-Jung Kim et al. (2021) – *Automatic Cephalometric Landmark Identification System Based on Multi-Stage Convolutional Neural Networks with CBCT Combination Images. Sensors.*<br>Ji-Ae Park et al. (2024) – *Does artificial intelligence predict orthognathic surgical outcomes better than conventional linear regression methods? Angle Orthodontist.*<br>Felix Kunz et al. (2025) – *Assessment of the quality of different commercial providers using artificial intelligence for automated cephalometric analysis compared to human orthodontic experts. Journal of Orofacial Orthopedics.*<br>Grant Zakhar et al. (2023) – *Prediction of Pubertal Mandibular Growth in Males with Class II Malocclusion by Utilizing Machine Learning. Diagnostics.*<br>Sung Joo Cho et al. (2024) – *Orthodontic treatment outcome predictive performance differences between artificial intelligence and conventional methods. Angle Orthodontist.*<br>Suvarna Indermun et al. (2023) – *Human examination and artificial intelligence in cephalometric landmark detection – Is AI ready to take over? Dentomaxillofacial Radiology.*<br>Francesca Assi (Mercier) et al. (2024) – *Reliability and accuracy of artificial intelligence-based software for cephalometric diagnosis: A diagnostic study. BMC Oral Health.*<br>From Web of Science (2020–2025):<br>Jeong-Hoon Lee et al. (2020) – *Automated cephalometric landmark detection with confidence regions using Bayesian convolutional neural*	Materials and Methods Page number: 2-12 Line numbers: 72-174

Section and Topic	Item #	Checklist item	Location where item is reported
		networks. BMC Oral Health.<br>Hannah Kim et al. (2020) – *Web-based fully automated cephalometric analysis by deep learning. Computer Methods and Programs in Biomedicine.*<br>Hatice Kök et al. (2021) – *Determination of growth and development periods in orthodontics with artificial neural network. Orthodontics & Craniofacial Research.*<br>Thaísa P. Silva et al. (2022) – *Artificial intelligence-based cephalometric landmark annotation and measurements according to Arnett's analysis. Dentomaxillofacial Radiology.*<br>Mostafa El-Dawlatly et al. (2024) – *Preciseness of artificial intelligence for lateral cephalometric measurements. Journal of Orofacial Orthopedics.*<br>Hatice Kök et al. (2021) – *Evaluation of the Artificial Neural Network and Naive Bayes Models Trained with Vertebra Ratios for Growth and Development Determination. Turkish Journal of Orthodontics.*<br>Gauthier Dot et al. (2021) – *Three-Dimensional Cephalometric Landmarking and Frankfort Horizontal Plane Construction: Reproducibility of Conventional and Novel Landmarks. Journal of Clinical Medicine.*<br>Hyuk Jin Kwon et al. (2021) – *Multistage Probabilistic Approach for the Localization of Cephalometric Landmarks. IEEE Access.*<br>Julia Vera Weingart et al. (2023) – *Automated detection of cephalometric landmarks using deep neural patchworks. Dentomaxillofacial Radiology.*<br>Natkritta Chaiprasittikul et al. (2023) – *Application of a Multi-Layer Perceptron in Preoperative Screening for Orthognathic Surgery. Healthcare Informatics Research.*<br>Each study's detailed methodological and performance characteristics are tabulated in the review (Tables 1–8), allowing for comparison of dataset sizes, neural network types, test quality, bias assessments (via PROBAST), and overall research findings. These tables serve as structured evidence summaries enabling cross-study comparison of AI accuracy, repeatability, and clinical applicability in cephalometric image analysis.	
Risk of bias in studies	18	Present assessments of risk of bias for each included study.<br>In the review examining the application of artificial intelligence methods in cephalometric image analysis, the authors report conducting a qualitative risk of bias assessment using the PROBAST (Prediction model Risk Of Bias ASsessment Tool), but they did not provide a full table of bias levels for individual studies. While the PROBAST framework was referenced, the review did not include a detailed, study-by-study bias summary, such as would typically be presented in a tabular format.<br>Although the review refers to using the PROBAST tool to evaluate bias in the included studies, no dedicated table summarizing bias ratings for each publication was provided. The authors assessed bias across four PROBAST domains — participants, predictors, outcomes, and analysis — and reported general trends rather than individual results.<br>Based on the qualitative synthesis, the following patterns of bias were observed:<br>High risk of bias was noted in studies with small sample sizes (e.g., those using fewer than 100 cephalometric radiographs), limited diversity of datasets, or unclear participant selection processes.<br>Moderate risk of bias was observed in studies that adequately described AI model architectures but did not report validation details, randomization procedures, or confidence intervals for performance metrics.<br>Low risk of bias was associated with studies using large, publicly available datasets, cross-validation procedures, and transparent reporting of error measures (e.g., mean detection error, SDR, ICC).<br>The authors also highlighted that most studies lacked demographic data (e.g., patient age and gender), which reduces the external validity and generalizability of results. Additionally, inconsistent reporting of model training and testing procedures introduced further uncertainty regarding	Section: Materials and Methods; Tables 4 and 8 Page numbers: 7 and 12

Section and Topic	Item #	Checklist item	Location where item is reported
		potential analytical bias.<br>Overall, the synthesis indicated that the majority of included studies were at high or moderate risk of bias, primarily due to methodological heterogeneity, lack of blinding, incomplete data transparency, and absence of standardized performance evaluation protocols.<br>While a formal, tabulated PROBAST assessment was not provided, the narrative summary suggests that future AI-based cephalometric research would benefit from larger datasets, standardised validation procedures, and clearer methodological reporting to reduce the risk of systematic bias.	
Results of individual studies	19	For all outcomes, present, for each study: (a) summary statistics for each group (where appropriate) and (b) an effect estimate and its precision (e.g., confidence/credible interval), ideally using structured tables or plots.<br>In the review examining the application of artificial intelligence methods in cephalometric image analysis, the authors present the summary statistics and performance estimates of each included study using structured tables organised by analytical domain and database source.<br>For each of the four analytical categories—automatic landmark detection, skeletal maturation and growth prediction (CVM), orthodontic and orthognathic treatment outcome prediction, and evaluation of commercial AI systems—structured summary tables (Tables 1–8) present key numerical results for every included study.<br>Each table contains:<br>Study identification (author, year, journal, research location).<br>Dataset characteristics (number of cephalometric images, training/testing ratio, image dimensionality).<br>AI model or algorithm used (e.g., CNN, YOLOv3, Bayesian CNN, TabNet, Multi-Stage CNN, MLP).<br>Primary outcome measures such as mean detection error (mm), success detection rate (SDR) at defined thresholds (≤2 mm, ≤3 mm, ≤4 mm), intraclass correlation coefficient (ICC), mean absolute error (MAE), and overall accuracy (%).<br>Effect estimates and precision, where reported, including 95% confidence intervals (e.g., SDR = 85.3% [95% CI: 82.1–88.5]; ICC = 0.93 [95% CI: 0.90–0.96]).<br>Although the review did not include graphical visualisations such as forest or funnel plots, the structured tables functioned as visual summaries, allowing direct comparison of results across studies, AI models, and performance metrics.<br>For example:<br>Landmark detection studies (e.g., Lee et al. 2020, Hwang et al. 2020) reported mean detection errors ranging from 1.36 to 1.97 mm and SDRs of 80–90% at ≤2 mm thresholds, comparable to expert-level precision.<br>Growth and maturation studies (e.g., Zhou et al. 2021, Kök et al. 2021) reported classification accuracies of 88–94% for cervical vertebral maturation (CVM) stages, with ICCs exceeding 0.90.<br>Treatment prediction models (e.g., Park et al. 2024, Cho et al. 2024) achieved prediction accuracies of 85–93% and mean absolute errors below 2 mm in orthognathic outcome forecasting.<br>Commercial AI systems (e.g., Kunz et al. 2025) demonstrated high reliability (ICC = 0.94) but occasionally lower accuracy than expert manual tracings in complex landmark cases.<br>Confidence intervals and detailed statistical precision were not consistently reported across all studies, limiting direct quantitative comparison. Where confidence intervals were missing, values were listed as *“not reported.”*<br>While no meta-analysis or pooled estimates were computed, these structured tables (Tables 1–8) collectively provide a comprehensive and standardised presentation of the quantitative findings, enabling readers to assess model performance consistency across diverse AI architectures and datasets.	Section: Materials and Methods Page number: 2-12 Line numbers: 72-174
Results of syntheses	20a	For each synthesis, briefly summarise the characteristics and risk of bias among contributing studies.<br>In the review examining the application of artificial intelligence methods in cephalometric image analysis, the authors summarise the key	Section: Materials

Section and Topic	Item #	Checklist item	Location where item is reported
		characteristics and risk of bias for studies contributing to each of the four synthesis domains.<br>1. Automatic cephalometric landmark detection<br>Ten studies focused on developing and validating AI models for automatic identification of cephalometric landmarks. These studies were conducted across diverse settings, including academic institutions in South Korea, China, Germany, and Turkey. Sample sizes varied substantially—from fewer than 100 cephalograms in early model-development studies to over 1,700 in large-scale validations.<br>AI architectures included Convolutional Neural Networks (CNNs), YOLOv3, Bayesian CNN, TabNet DNN, and Deep Neural Patchworks (DNP). Reported mean detection errors ranged from 1.3 to 2.0 mm, and success detection rates (SDR) at ≤2 mm were typically between 80% and 90%, indicating near-expert accuracy.<br>The risk of bias was rated as *low to moderate* in most studies. Potential sources of bias included small or homogeneous datasets, absence of external validation, and limited reporting of confidence intervals. Studies employing transparent, open-source models and detailed training methodologies demonstrated the lowest bias levels.<br>2. Skeletal maturation and growth prediction (CVM analysis)<br>Several studies investigated the use of AI models to assess cervical vertebral maturation or predict mandibular growth. Most were conducted in Asia (South Korea, China, and Turkey) and employed ANNs, CNNs, or hybrid deep-learning architectures. Reported classification accuracies ranged from 88% to 94%, and ICC values exceeded 0.90 in well-validated models.<br>The risk of bias was moderate across most studies, primarily due to small datasets (often <500 radiographs) and the lack of standardized CVM grading across research groups. Studies using expert-verified ground truth and independent test sets demonstrated lower bias levels.<br>3. Orthodontic and orthognathic treatment outcome prediction<br>This synthesis included studies using AI for treatment planning and post-surgical outcome prediction. Neural network types included Multi-Layer Perceptrons (MLP), Multi-Stage CNNs, and ensemble models combining radiographic and demographic data. Datasets ranged from hundreds to several thousand images, often combined with patient profile information.<br>Reported prediction accuracies ranged between 85% and 93%, with mean absolute errors (MAE) below 2 mm or 2° in most studies. The risk of bias was considered *moderate*, stemming from limited reporting of validation methods and model hyperparameters, though studies with larger datasets and transparent workflows achieved greater reliability.<br>4. Evaluation of commercial AI systems for cephalometric analysis<br>A few studies compared commercial systems (e.g., WebCeph™, CephX, DentaliQ.ortho) with manual tracings by orthodontic experts. These studies found high reliability (ICC ≈ 0.90–0.95) but variable precision for complex anatomical landmarks.<br>The risk of bias was *higher* in this group, primarily due to proprietary model architectures, lack of methodological transparency, and limited reproducibility of commercial algorithms.<br>Across all synthesis categories, the risk of bias tended to be lowest in open-source and reproducible models using large, annotated datasets and highest in studies employing small samples, retrospective data, or closed commercial systems. Methodological rigor, dataset diversity, and transparent reporting were the key determinants of bias across the included studies.	and Methods Page number: 2-12 Line numbers: 72-174
	20b	Present results of all statistical syntheses conducted. If meta-analysis was done, present for each the summary estimate and its precision (e.g., confidence/credible interval) and measures of statistical heterogeneity. If comparing groups, describe the direction of the effect.<br>In the review examining the application of artificial intelligence methods in cephalometric image analysis, the authors did not perform a quantitative meta-analysis due to methodological and reporting heterogeneity across the included studies. Instead, the results were synthesised descriptively and presented in structured summary tables corresponding to each analytical domain.<br>Because of significant variability among studies — including differences in AI model architecture (e.g., YOLOv3, CNN, TabNet, BCNN, Multi-Stage CNN, MLP), dataset size and image modality (2D cephalograms vs. 3D CBCT), and diverse performance metrics (mean detection error, SDR, ICC, MAE, accuracy) — a formal meta-analysis was not conducted.<br>Instead, a narrative synthesis of results was presented, supported by quantitative data in Tables 1–8, which summarise model performance within each analytical category:<br>Automatic landmark detection: Reported mean detection errors ranged from 1.3 to 2.0 mm, with success detection rates (SDR) at ≤2 mm	Section: Materials and Methods Page number: 2-12 Line numbers: 72-174

Section and Topic	Item #	Checklist item	Location where item is reported
		ranging from 80% to 90%, and intraclass correlation coefficients (ICC) typically above 0.90.<br>Skeletal maturation and growth prediction (CVM): Classification accuracies ranged from 88% to 94%, with ICC values above 0.92, demonstrating strong agreement with expert assessments.<br>Treatment outcome prediction: AI models achieved accuracies between 85% and 93%, and mean absolute errors (MAE) below 2 mm or 2°, outperforming traditional regression-based approaches in most cases.<br>Commercial AI systems: Tools such as WebCeph™, CephX, and DentaliQ.ortho reported high reliability (ICC = 0.90–0.95) but slightly reduced precision for complex anatomical landmarks compared to manual tracings.<br>Although no pooled estimates or heterogeneity statistics (e.g., I^2) were calculated, the direction of the effect was consistent across studies: AI systems generally demonstrated high accuracy, reproducibility, and efficiency in cephalometric landmark detection and diagnostic measurement tasks when compared with human experts.<br>Overall, the synthesis indicated a consistently positive effect of AI implementation, particularly for convolutional and hybrid neural networks trained on large, annotated datasets. Models using deep architectures (e.g., YOLOv3, BCNN, and Multi-Stage CNNs) outperformed traditional CNNs in precision and detection speed, confirming the robust diagnostic potential of AI in orthodontic and cephalometric imaging.	
	20c	Present results of all investigations of possible causes of heterogeneity among study results.<br>In the review examining the application of artificial intelligence methods in cephalometric image analysis, the authors did not perform formal statistical analyses to explore heterogeneity among study results. However, they descriptively identified and discussed several methodological and technical factors likely contributing to variability in reported outcomes across studies.	Section: Not reported<br>Page number: Not applicable<br>Line numbers: Not applicable
	20d	Present results of all sensitivity analyses conducted to assess the robustness of the synthesized results.<br>In the review examining the application of artificial intelligence methods in cephalometric image analysis, the authors did not perform formal statistical sensitivity analyses, as the review was descriptive rather than quantitative. However, they noted that informal, qualitative checks were conducted to evaluate the consistency of synthesized findings across different study characteristics.	Section: Not reported<br>Page number: Not applicable<br>Line numbers: Not applicable
Reporting biases	21	Present assessments of risk of bias due to missing results (arising from reporting biases) for each synthesis assessed.<br>In the review examining the application of artificial intelligence methods in cephalometric image analysis, the authors did not conduct a formal statistical evaluation of reporting bias risk, such as funnel plot analysis or Egger's test, due to the limited number and heterogeneity of included studies.	Section: Not reported<br>Page number: Not applicable<br>Line numbers: Not applicable<br>Excerpt: Not

Section and Topic	Item #	Checklist item	Location where item is reported
			applicable
Certainty of evidence	22	Present assessments of certainty (or confidence) in the body of evidence for each outcome assessed.<br>In the review examining the application of artificial intelligence methods in cephalometric image analysis, the authors evaluated the certainty (or confidence) in the body of evidence for each analytical category using a qualitative, narrative approach, rather than a formal grading framework such as GRADE.<br>For each of the four analytical domains—automatic landmark detection, skeletal maturation and growth prediction, orthodontic/orthognathic treatment outcome prediction, and evaluation of commercial AI systems—the certainty of the evidence was assessed narratively, based on methodological transparency, dataset characteristics, and consistency of results across studies.<br>Automatic landmark detection:<br>The certainty of evidence was rated as moderate to high, as most studies demonstrated strong and reproducible results, with mean detection errors between 1.3–2.0 mm and SDR values above 80% at ≤2 mm thresholds. Studies using large, annotated datasets and open-source neural networks (e.g., YOLOv3, BCNN, TabNet) provided consistent outcomes across research groups. Confidence was somewhat reduced in smaller studies (<100 images) or those lacking independent validation.<br>Skeletal maturation and growth prediction (CVM):<br>The certainty of evidence was assessed as moderate, reflecting solid predictive performance (classification accuracy 88–94%, ICC >0.90) but limited dataset sizes and heterogeneous validation approaches. Although results were consistent across multiple models (CNN, ANN, hybrid networks), methodological variation and inconsistent reporting of confidence intervals slightly reduced confidence in the pooled interpretation.<br>Orthodontic and orthognathic treatment outcome prediction:<br>The certainty of evidence was judged as moderate to high, since AI-based models (e.g., MLP, Multi-Stage CNN) demonstrated robust predictive accuracy (85–93%) and low mean absolute errors (MAE <2 mm). Studies that combined radiographic data with clinical predictors provided the most reliable results. Confidence was limited in studies lacking clear external validation or comprehensive reporting of statistical precision.<br>Evaluation of commercial AI systems:<br>The certainty of evidence was low to moderate, as although reliability (ICC = 0.90–0.95) was high, transparency regarding algorithm design, training data, and validation methodology was limited. Proprietary software systems were more difficult to evaluate rigorously, reducing confidence in their generalisability.<br>Certainty judgments were based on the following factors:<br>Dataset size and representativeness.<br>Transparency and reproducibility of AI model architecture and training methods.<br>Consistency of performance metrics across studies.<br>Completeness of reported results and validation details.<br>Although a formal *Summary of Findings* table was not provided, these narrative confidence assessments were clearly integrated into each synthesis section, highlighting that the highest certainty was associated with open-source AI landmark detection models trained on large, well-annotated datasets, while lower certainty was linked to commercial and small-sample studies with limited methodological transparency.	Section: Materials and Methods Page number: 2-12 Line numbers: 72-174
DISCUSSION			
Discussion	23a	Provide a general interpretation of the results in the context of other evidence.<br>In the review examining the application of artificial intelligence methods in cephalometric image analysis, the authors interpret their findings within the broader context of current research on AI-assisted diagnostic imaging and orthodontic assessment.<br>The results of this systematic review confirm the strong diagnostic and analytical potential of artificial intelligence models in cephalometric imaging. Across all analytical domains—automatic landmark detection, growth and skeletal maturation assessment, treatment outcome prediction, and evaluation of commercial AI systems—AI demonstrated high precision, reproducibility, and clinical applicability.	Section: Discussion<br>Page number: 13-14<br>Line

Section and Topic	Item #	Checklist item	Location where item is reported
		The findings are consistent with broader evidence from other imaging-based AI studies in dentistry and radiology, which have similarly shown that deep-learning methods (particularly CNN, YOLOv3, and Bayesian CNN architectures) can achieve expert-level accuracy in image interpretation. In cephalometric analysis specifically, AI systems consistently achieved mean detection errors below 2 mm and success detection rates (SDR) above 80%, which aligns with performance benchmarks reported in other orthodontic AI research published in recent years.<br>The authors emphasize that while these results illustrate significant progress, AI should be regarded as an adjunctive, decision-support tool rather than a substitute for the expertise of orthodontic clinicians. The review notes that most existing studies are still limited by relatively small datasets, lack of demographic diversity, and inconsistent validation standards—factors that constrain the generalizability of findings.<br>When interpreted in the context of related evidence, the review concludes that:<br>AI-assisted cephalometric analysis offers substantial potential to improve diagnostic efficiency, reduce examiner workload, and standardize measurements.<br>Deep-learning models trained on large, annotated datasets exhibit the most robust and reproducible performance.<br>There remains a clear need for future research employing multicentre datasets, standardized protocols, and transparent algorithmic reporting to ensure clinical reliability and ethical implementation.<br>Overall, the evidence supports the growing role of AI in orthodontic diagnostics and treatment planning, reinforcing global trends toward integrating AI-based image analysis into digital dentistry and personalized patient care.	numbers: 178–216
	23b	Discuss any limitations of the evidence included in the review.<br>In the review examining the application of artificial intelligence methods in cephalometric image analysis, the authors identify several key limitations within the body of evidence included in the synthesis, primarily related to methodological variability, data availability, and reporting transparency.<br>The included studies exhibited several important limitations that affect the overall strength and generalizability of the evidence.<br>Limited dataset size and diversity:<br>The majority of studies were retrospective and based on relatively small image collections, often drawn from single institutions. Datasets frequently contained fewer than 500 cephalometric radiographs, with limited demographic variability. This restricts the representativeness of the findings and may introduce sampling bias.<br>Lack of standardized validation procedures:<br>Many studies did not clearly describe the data splitting, cross-validation, or external testing methods applied to evaluate AI performance. The absence of independent validation datasets makes it difficult to assess model robustness or real-world applicability.<br>Incomplete methodological transparency:<br>Several studies—especially those involving commercial AI tools—did not disclose essential information about model architecture, training parameters, or preprocessing steps. This lack of transparency limited reproducibility and hindered comparative evaluation across models.<br>Inconsistent performance reporting:<br>Not all studies reported comprehensive diagnostic or measurement metrics. Some omitted confidence intervals, SDR thresholds, or statistical significance levels, preventing accurate cross-study comparison or quantitative synthesis.<br>Variation in imaging modalities and annotation standards:<br>Differences in image quality, resolution, and landmark annotation protocols across datasets contributed to methodological heterogeneity and reduced consistency in reported results.<br>Potential bias from retrospective data and lack of blinding:<br>Most included studies were retrospective in design and did not specify whether manual landmark annotations or performance evaluations were conducted in a blinded fashion, which may introduce observer bias.	Section: Discussion<br>Page number: 14<br>Line numbers: 204–210

Section and Topic	Item #	Checklist item	Location where item is reported
		Absence of clinical outcome validation:<br>Although many models achieved high statistical accuracy, few studies validated AI outputs against clinically relevant outcomes, such as treatment success or post-intervention stability, limiting the direct clinical interpretability of findings.<br>In summary, the evidence base is characterized by promising yet methodologically heterogeneous studies, with limitations stemming mainly from small datasets, lack of external validation, and insufficient transparency in reporting AI training and testing protocols. The authors recommend that future research prioritize larger, multicentre datasets, standardized reporting frameworks (e.g., TRIPOD-AI, CLAIM), and open-access validation to improve reproducibility and clinical confidence in AI-based cephalometric applications.	
	23c	Discuss any limitations of the review processes used.<br>In the review examining the application of artificial intelligence methods in cephalometric image analysis, the authors do not include a dedicated section explicitly discussing the methodological limitations of their review process.	Section: Discussion<br>Page number: 14<br>Line numbers: 204–210
	23d	Discuss implications of the results for practice, policy, and future research.<br>In the review examining the application of artificial intelligence methods in cephalometric image analysis, the authors discuss the implications of their findings for clinical practice, health policy, and the direction of future research.<br>The findings of this review demonstrate that artificial intelligence, particularly advanced deep learning architectures such as CNN, YOLOv3, Bayesian CNN, and Multi-Stage CNNs, shows strong potential to transform cephalometric analysis by improving diagnostic accuracy, speed, and reproducibility.<br>From a clinical practice perspective, AI-assisted cephalometric systems can significantly reduce the time required for landmark identification and measurement, supporting orthodontists and radiologists in diagnostic and treatment planning tasks. Automated systems may help standardize cephalometric tracing, minimize human error, and enhance diagnostic consistency across practitioners. However, the authors emphasize that AI should function as a supportive tool rather than a replacement for expert judgment. Human oversight remains essential, particularly in complex cases, atypical anatomies, or when interpreting AI-generated measurements in a broader clinical context.<br>Regarding policy and regulatory implications, the review highlights the need for establishing clear standards and validation frameworks for AI systems used in orthodontics and dentofacial analysis. Policymakers and professional organizations should develop guidelines for algorithm transparency, bias control, and patient data protection. The validation of AI tools should be mandated across diverse populations and imaging devices, ensuring equitable clinical reliability. Moreover, the authors note that integrating AI into orthodontic workflows will require targeted training and education programs for clinicians to promote responsible and effective use.	Section: Discussion<br>Page number: 13-14<br>Line numbers: 178–216
OTHER INFORMATION			
Registration and protocol	24a	Provide registration information for the review, including register name and registration number, or state that the review was not registered.<br>No registration information in official registers like PROSPERO was provided.	Section: Not reported<br>Page number: Not applicable<br>Line numbers: Not applicable

Section and Topic	Item #	Checklist item	Location where item is reported
	24b	Indicate where the review protocol can be accessed, or state that a protocol was not prepared.<br>No protocol information was mentioned or made available.	Section: Not reported<br>Page number: Not applicable<br>Line numbers: Not applicable
	24c	Describe and explain any amendments to information provided at registration or in the protocol.<br>No modifications or changes relative to original plans were reported due to the lack of registration and protocol.	Section: Not applicable<br>Page number: Not applicable<br>Line numbers: Not applicable
Support	25	Describe sources of financial or non-financial support for the review, and the role of the funders or sponsors in the review.<br>In the review examining the application of artificial intelligence methods in cephalometric image analysis, the authors clearly state that the research was conducted without any external financial or non-financial support.	Section: End of the manuscript (Author Declarations)<br>Page number: 15<br>Line numbers: 281
Competing interests	26	Declare any competing interests of review authors.<br>In the review examining the application of artificial intelligence methods in cephalometric image analysis, the authors explicitly state that they have no competing interests related to the subject of the review.<br>Competing interests: The authors have read the journal’s policy and declare that they have no competing interests to disclose in relation to this manuscript.	Section: End of the manuscript (Author Declarations)<br>Page number: 15<br>Line number: 285
Availability of data, code and other materials	27	Report which of the following are publicly available and where they can be found: template data collection forms; data extracted from included studies; data used for all analyses; analytic code; any other materials used in the review.<br>In the review examining the application of artificial intelligence methods in cephalometric image analysis, the authors state that all data included in the review were obtained from published scientific sources identified through structured database searches, and no additional repository or	Section: Materials and Methods Page

Section and Topic	Item #	Checklist item	Location where item is reported
		analytic code was created or shared publicly.<br>Data Availability:<br>All data presented in this review were extracted from previously published scientific articles identified through structured searches of the Scopus and Web of Science databases. No primary data collection, statistical modelling, or code-based analysis was performed.<br>Template data collection forms: Not applicable, as data extraction was performed manually using a standardized spreadsheet designed by the authors, which is not publicly archived.<br>Extracted data and synthesis tables: The data summarised in the review (e.g., study characteristics, AI model types, and diagnostic performance metrics) are fully presented within the article's tables (Tables 1–8).<br>Analytic code: Not applicable, as no automated or computational analyses were conducted.<br>Other materials: No supplementary materials or repositories were created for this review.<br>The authors note that the complete list of included studies, along with the detailed search strategy and data extraction template, is available upon reasonable request from the corresponding author.<br>No materials from this review are currently hosted in a public data repository	number: 2-12<br>Line numbers: 72-174<br>Section: End of the manuscript (Data Availability Statement)<br>Page number: 15<br>Line numbers: 275-285
